# Deep Learning for Classifying and Cognitive Profiling of Subcortical Vascular Cognitive Impairment

**DOI:** 10.34133/cbsystems.0561

**Published:** 2026-05-13

**Authors:** Miao He, Yunsi Yin, Junda Qu, Yan Wang, Xinwei Que, Xinyi Xia, Tongtong Zhang, Jiangting Li, Junyi Shen, Weihong Song, Qi Qin, Chunlin Li, Yi Tang

**Affiliations:** ^1^ School of Biomedical Engineering, Capital Medical University, Beijing 100069, China.; ^2^ Department of Neurology & Innovation Center for Neurological Disorders, Xuanwu Hospital, Capital Medical University, National Center for Neurological Disorders, Beijing 100053, China.; ^3^ Department of Radiology & Precision and Intelligence Medical Imaging Lab, Beijing Friendship Hospital, Capital Medical University, Beijing 100050, China.; ^4^ Center for Geriatric Medicine, International Center for Alzheimer’s Research, Prevention and Treatment, The First Affiliated Hospital and Oujiang Laboratory; Key Laboratory of Alzheimer’s Disease of Zhejiang Province, Institute of Aging, Wenzhou Medical University, Wenzhou, Zhejiang 325000, China.

## Abstract

Subcortical vascular cognitive impairment (SVCI) is a heterogeneous cognitive impairment caused by small vessel disease. Diagnosis of SVCI remains challenging when neuropsychological assessment is impractical. This study proposes a diffusion tensor imaging (DTI)-based DenseNet to identify SVCI from subcortical ischemic vascular disease (SIVD) and to profile multidomain cognitive risks. We collected neuropsychological scales and DTI from 134 SVCI and 171 SIVD patients in our internal dataset for model development. An external target-domain dataset of 90 SVCI and 103 SIVD patients was used for unsupervised domain adaptation (UDA). Within this dataset, 45 SVCI and 53 SIVD patients were used for unlabeled UDA fitting; the remaining 45 SVCI and 50 SIVD patients were held out as a target-domain test set. Model-generated salient maps identified white matter (WM) regions associated with SVCI. Mutual information (MI) maps between DTI and 6 neuropsychological scales were computed to identify structural correlates of cognitive domains for cognitive profiling. We computed structural similarity index measure (SSIM) between individual-level salient maps derived from DenseNet and the MI maps for unsupervised clustering to stratify domain-specific cognitive impairment risk in SVCI. The DenseNet achieves high accuracy (0.902 internal, 0.926 target-domain) with AUCs of 0.951 and 0.942, respectively. SVCI probabilities reflect cognitive severity, and salient maps are associated with neuropsychological performance. Regarding cognitive profiling, each cognitive domain is divided into low, moderate, and high subgroups, with significantly different SSIM. Our DTI-based study demonstrates accurate SVCI identification and individualized multi-domain cognitive profiling. This offers a complementary framework to support diagnosis and personalized intervention.

## Introduction

Subcortical ischemic vascular disease (SIVD) is pathologically driven by stenosis and occlusion of small vessels, resulting in white matter hyperintensities (WMHs) and multiple lacunar infarcts [1]. The prevalence of SIVD rises markedly with advancing age, affecting about 5% of individuals around 50 years of age, and is almost ubiquitous in populations over 90 years [2]. Approximately 50% of SIVD patients exhibit progressive cognitive deterioration and eventually convert to subcortical vascular cognitive impairment (SVCI) [3–5]. This constitutes a significant burden in aging populations [5–7]. Clinically, identifying SVCI from SIVD is essential for initiating early intervention, which holds the potential to slow cognitive decline and improve long-term outcomes. Notably, there is an increasing recognition that cognitive impairment in SVCI is heterogeneous, affecting distinct cognitive domains across individuals, such as memory, executive function, attention, and visuospatial processing [8,9]. A precise delineation of domain-specific cognitive impairments at the individual level is crucial for personalized intervention and risk stratification.

According to the latest Vascular Impairment of Cognition Classification Consensus Study guidelines, the diagnosis of SVCI relies on multimodal assessments of clinical symptoms, structural magnetic resonance imaging (MRI), and neuropsychological scale [10,11]. However, this multimodal diagnostic approach is often impractical in elderly or resource-limited settings. Comprehensive neuropsychological assessments involving Mini-Mental State Examination (MMSE), Montreal Cognitive Assessment (MoCA), Immediate Recall, Delayed Recall, Trail Making Test-A (TMT-A), and Trail Making Test-B (TMT-B) typically require up to 60 min to administer and are vulnerable to evaluator bias, which induces the risk of misdiagnosis [12–14]. Therefore, the current challenge lies in developing more accessible, objective, and scalable methods for identifying SVCI from SIVD and performing domain-specific cognitive profiling at the individual level. In this context, imaging-based automated approaches may serve as a complementary solution that can support SVCI differentiation and cognitive profiling, particularly valuable in cases where comprehensive neuropsychological assessments are unavailable or infeasible.

Structural MRI is widely used to assess brain structural abnormalities in SIVD patients [15–18]. On T2-weighted and fluid-attenuated inversion recovery (FLAIR) images, SIVD and SVCI typically demonstrate asymptomatic lacunes infarcts, and WMHs [19–21]. However, WMHs are also commonly observed in asymptomatic elderly populations, with rates rising to approximately 95% by age 80 [22]. High baseline prevalence limits the specificity of WMH burden in identifying SVCI from SIVD. Moreover, the relationship between WMH severity and the degree of cognitive impairment is still uncertain [23–25]. These findings suggest that structural MRI, although accessible and informative, may lack the sensitivity and specificity needed to detect subtle microstructural changes underlying cognitive decline in SVCI. Benefiting from advances in neuroimaging and MRI, diffusion tensor imaging (DTI) has been widely applied to assess microstructural abnormalities in white matter (WM) and provides unique information that conventional structural MRI cannot offer [26]. Studies have shown that specific WM damage on DTI images correlates with neuropsychological deficits in SVCI [27,28]. These findings highlight the potential of DTI to reveal the neuroimaging signatures in SVCI [29–31].

The advancement of artificial intelligence offers opportunities to support and improve SVCI diagnosis. Studies applying machine learning approaches have demonstrated the feasibility of automated classification. However, these studies (including our previous study) rely heavily on handcrafted feature engineering to uncover potential discriminators [32–37]. Such dependence introduces variability and bias that constrain their diagnostic performance and out-domain performance. Deep learning offers a powerful alternative by directly learning hierarchical features from raw imaging data. Recent studies have demonstrated promising results for SVCI diagnosis [38–40]. However, their clinical translation remains limited due to small sample sizes, lack of external validation, and poor interpretability. Most existing VCI models are trained and evaluated under data-scarce conditions, raising concerns about overfitting and the restricted out-domain performance of the models. Unsupervised domain adaptation (UDA) techniques may provide a solution to tackle the performance degradation of deep learning models when being deployed to an unseen dataset [41,42]. Despite these methodological advances, current studies only focus on SVCI diagnosis, overlooking the multidimensional nature of cognitive impairment in SVCI patients. Consequently, existing studies lack the capacity to capture cognitive domain heterogeneity, limiting their relevance for individualized diagnosis or intervention planning. Whether deep learning can move beyond SVCI diagnosis to support multidomain cognitive profiling at the individual level based on neuroimaging data remains a question. A solution to this could enable more personalized cognitive risk stratification and targeted intervention, promoting precision medicine in SVCI.

In the present study, we constructed the DenseNet model with the UDA strategy to identify SVCI from SIVD patients solely based on DTI data and to analyze multidomain cognitive profiles of SVCI patients. Compared with our previous study on SVCI [32], the present study made methodological and conceptual advances. Our previous study relied on a multimodal machine learning pipeline integrating handcrafted features derived from T1-weighted imaging, DTI, and resting-state functional MRI. The present study adopted single-modal deep learning model to eliminate manual feature engineering and multimodal data acquisition requirements. This reduced data dependency and improved methodological accessibility and clinical applicability. We incorporated a UDA strategy to explicitly address intersite distribution shifts. This enables the model to learn domain-invariant imaging representations, thereby improving robustness and out-of-domain performance. We evaluated the constructed DenseNet model on both internal and target-domain test sets and demonstrated better performance than our previous multimodal machine learning method. Additionally, we visualized salient regions for the DenseNet model as SVCI risk brain regions and evaluated their neuropsychological relevance. Conceptually, our cognitive profiling approach moves beyond binary SVCI identification toward assessment of cognitive impairment heterogeneity at the individual level. We integrated model-derived salient maps with neuropsychological spatially mutual information (MI) maps to construct a multidomain cognitive profiling approach. For SVCI patients, we used a model-generated salient weight map to assess the cognitive impairment risk of 6 cognitive domains. This approach provided an imaging-based solution for personalized and comprehensive neuropsychological assessment, offering insights to guide cognitive intervention strategies.

## Materials and Methods

### Experimental design

**Fig. 1.**
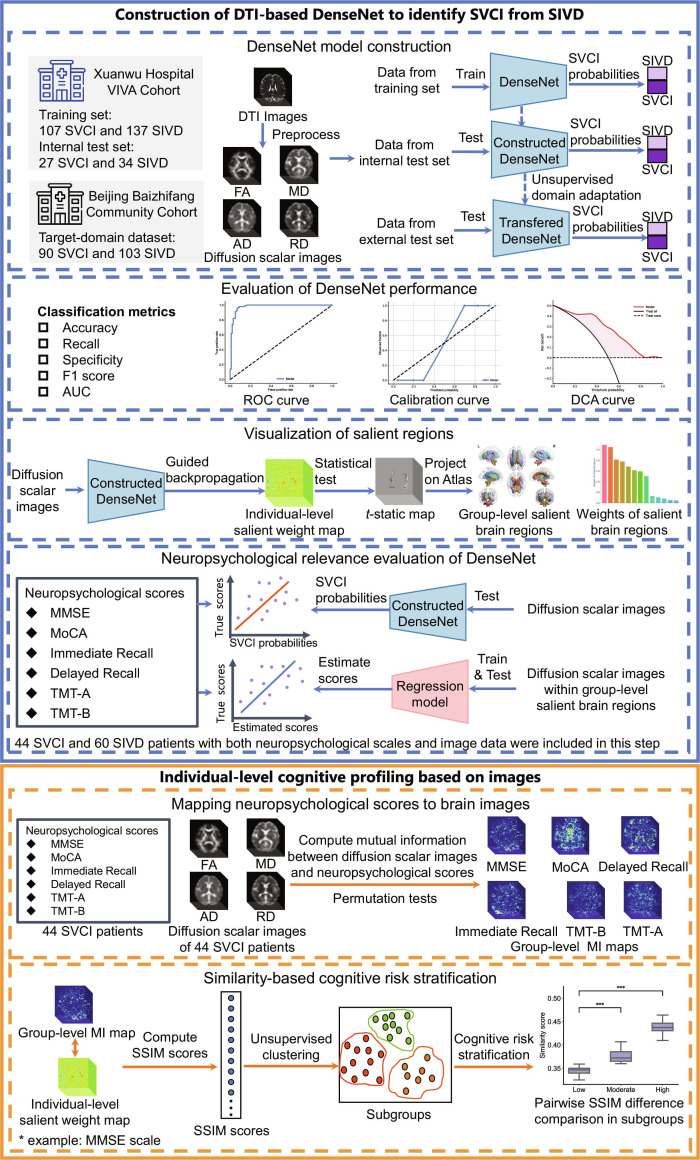
Flowchart of the study. The workflow consists of 2 major steps. The blue panel depicts the construction of a diffusion tensor imaging (DTI)-based DenseNet model to identify subcortical vascular cognitive impairment (SVCI) from subcortical ischemic vascular disease (SIVD) patients using diffusion scalar image combinations. Diffusion scalar images were derived from preprocessed DTI images. An unsupervised domain adaptation strategy was applied to enhance the model’s performance on unseen target-domain data, and model performance was evaluated on both internal and target-domain test sets. The neuropsychological relevance of model outputs—including SVCI probability and salient white matter regions—was further assessed. The orange panel illustrates the individual-level cognitive profiling based on images. Voxel-wise mutual information (MI) maps were computed between diffusion scalar images and 6 neuropsychological scales to identify domain-specific white matter correlates, and structural similarity index measure (SSIM) was calculated between individual-level model-derived salient weight maps and MI maps. Unsupervised clustering of SSIM scores enabled imaging-based cognitive risk stratification across cognitive domains. FA, fractional anisotropy; MD, mean diffusivity; AD, axial diffusivity; RD, radial diffusivity; ROC, receiver operating characteristic; AUC, area under the curve; DCA, decision curve analysis; MMSE, Mini-Mental State Examination; MoCA, Montreal Cognitive Assessment; TMT-A, Trail Making Test-A; TMT-B, Trail Making Test-B.

The workflow of the present study consists of 2 major components, as shown in Fig. 1. First, a DTI-based DenseNet model was constructed to identify SVCI from SIVD patients, incorporating a UDA strategy to improve model’s performance on unseen target-domain data. Model performance was evaluated on both internal and target-domain test sets, and its neuropsychological relevance was assessed. Second, we performed individual-level cognitive profiling based on images. We computed voxel-wise MI maps between DTI-derived diffusion scalar images and 6 neuropsychological scales (MMSE, MoCA, Immediate Recall, Delayed Recall, TMT-A, TMT-B). These MI maps reflect the group-level structural correlates of each cognitive domain. We quantified the spatial similarity between each patient’s salient weight map derived from the DenseNet model and the MI maps for each cognitive scale using the structural similarity index measure (SSIM). SSIM scores capture how closely an individual’s WM alteration pattern aligns with domain-specific neuroanatomical relevance. Finally, for each cognitive domain, patients were clustered using *K*-means based on their SSIM scores. The resulting clusters—interpreted as low, moderate, and high risk—represent different levels of cognitive impairment likelihood in the corresponding domain. Statistical comparisons were performed to confirm whether there were significant differences in SSIM scores between clusters. This framework enables imaging-based cognitive risk stratification across multiple domains at the individual level

### Participants

The internal dataset was retrospectively collected from the Vascular Impairment and Vascular Aging (VIVA) cohort of Capital Medical University Xuanwu Hospital, including 134 patients who met the inclusion criteria for SVCI and 171 patients with SIVD without cognitive impairment. For UDA of deep learning model, a target-domain dataset was collected from the Beijing Baizhifang community, consisting of 90 SVCI patients and 103 age-matched SIVD patients without cognitive impairment, selected by the same inclusion criteria (Fig. S1). The ethics committee of Xuanwu Hospital approved this study. Written informed consent according to the Declaration of Helsinki was provided by all participants.

The diagnosis of SVCI was made by 3 senior neurologists, and each patient met the following inclusion criteria: (a) age 50 to 85; (b) complaint and/or informant report of cognitive impairment for at least 3 months; (c) met the Diagnostic and Statistical Manual of Mental Disorders 5th Edition criteria for cognitive impairment, including a Clinical Dementia Rating (CDR) ≥ 0.5 on at least one domain and a global score ≥ 0.5; and (d) MRI with (i) moderate to severe WM lesions (Fazekas score ≥ 2), or multiple (≥3) small supratentorial subcortical infarcts (3 to 20 mm in diameter), or small infarcts strategically located in the caudate nucleus, globus pallidus, or thalamus; (ii) absence of hemorrhages, cortical and watershed infarcts, hydrocephalus, and WM lesions from specific causes; and (iii) no hippocampal or entorhinal cortex atrophy (score = 0, according to the medical temporal lobe atrophy score).

The inclusion criteria for patients with SIVD without cognitive impairment were as follows: (a) age 50 to 85 years; (b) evidence of relevant subcortical vascular ischemia by MRI, including (i) moderate to severe WM lesions (Fazekas score ≥ 2); multiple (≥3) small supratentorial subcortical infarcts (3 to 20 mm in diameter), or small infarcts strategically located in the caudate nucleus, globus pallidus, or thalamus; (ii) absence of hemorrhages, cortical and watershed infarcts, hydrocephalus, and WM lesions from specific causes; (iii) no hippocampal or entorhinal cortex atrophy (score = 0, according to the medical temporal lobe atrophy score); (c) no complaint and/or other cognitive domains; and (d) CDR score = 0 for every domain.

Patients with the following conditions were excluded: (a) other cognitive impairments or neurological disease; (b) any condition, including illiteracy, hearing disorder, or visual impairment, that would preclude completion of neuropsychological assessment testing; and (c) diagnosis of Alzheimer’s disease by plasma amyloid β 1–42 and/or phosphorylated tau 181 concentrations after 2 years of follow-up.

### Image acquisition and preprocessing

All images were acquired using Siemens 3T MRI scanner with a 32-channel head coil for head scanning. Stuffing was placed to avoid participant head movement. DTI images were acquired using a diffusion-weighted double spin-echo echo-planar imaging (EPI) sequence: repetition time (TR), 8,000 ms; echo time (TE), 96 ms; 64 diffusion-weighted directions with a *b* value of 1,000 s/mm^2^ and 11 images with a *b* value of 0 s/mm^2^; flip angle, 90°; field of view (FOV), 224 mm × 224 mm; matrix, 128 × 128, in-plane resolution, 1.75 mm × 1.75 mm voxels; 60 contiguous 2-mm-thick axial slices.

The raw DTI images were preprocessed using the Pipeline for Analyzing braiN Diffusion imAges (PANDA, www.nitrc.org/projects/panda) toolbox to obtain the diffusion scalar images: fractional anisotropy (FA), mean diffusivity (MD), axial diffusivity (AD), and radial diffusivity (RD). All images were spatially normalized to the Montreal Neurological Institute (MNI) space. To reduce scanner-related bias in multi-center data from internal and external cohorts, we applied the Combating Batch Effects (Combat) data harmonization method implemented in the Neuroharmony tool [43]. Combat parameters were fitted exclusively on the training data and then applied to the test set.

### DenseNet model construction

The DenseNet [44] model was constructed to identify SVCI from SIVD patients. The model architecture is illustrated in Fig. S2A. The model consists of 4 dense blocks, 3 transition layers, one global average pooling layer, and one fully connected layer. Each dense block contains multiple dense layers, where each dense layer is composed of 2 convolutional layers with BatchNorm and ReLU activation functions. In the present study, the constructed model has one dense layer in each dense block. The transition layers include one convolutional layer with BatchNorm and ReLU, followed by an average pooling layer with a stride of 2, which is used to down-sample the feature maps. The first convolution layer has 64 filters, and each subsequent layer increases by 32 filters. The SVCI probability was predicted at the end of the model by a softmax function. To avoid overfitting, a dropout rate of 0.5 was applied before the fully connected layer.

Twenty percent of the internal dataset was randomly assigned as the internal test set, while the remaining 80% was used as the training set for 5-fold cross-validation during model training. Following cross-validation, the optimal training parameters were identified and used to retrain the model on the full training set.

During model training, on-the-fly data augmentation was performed using intensity and spatial transformations. The augmentation pipeline included randomly shifting intensity from 0.9 to 1.1 or randomly scaling intensity to 1.1 times with a probability of 30%, flipping the image along the first axis, or randomly zooming the image range from 0.9 to 1.1 with a probability of 20%. Intensity normalization was performed separately on training and test sets to ensure no data leakage. During the transforms pipeline, parameters were computed and applied independently for each sample within each set so that training and test data were processed separately. The detailed augmentation summary is shown in Table S1. To compare the impact of data augmentation, we also trained DenseNet models without data augmentation. The model was trained from scratch with a batch size of 16 and a learning rate of 0.00005 for 50 epochs. The proposed DenseNet model contains 523,034 parameters, and the GPU memory usage was 560.57 MB. The training of the DenseNet model takes approximately 29.30 min on a workstation equipped with an NVIDIA Quadro RTX 8000 GPU. A cosine annealing algorithm was employed to dynamically adjust the learning rate. Cross-entropy loss was selected as the loss function, and the adaptive moment estimation (Adam) optimizer was used to optimize the model’s parameters with weight decay set at 0.001.

To find the optimal DTI data combination to identify SVCI, the following 15 DTI data combinations were used to train the model: (a) FA; (b) MD; (c) AD; (d) RD; (e) FA and MD; (f) MD and AD; (g) AD and RD; (h) FA and AD; (i) MD and RD; (j) FA and RD; (k) FA, MD, and AD; (l) MD, AD, and RD; (m) FA, AD, and RD; (n) FA, MD, and RD; (o) FA, MD, AD, and RD. In DTI data combinations with more than one diffusion scalar image, multimodal diffusion scalar images were concatenated at the channel dimension before being fed into the model.

### UDA of DenseNet

To enhance the performance of our DenseNet model across heterogeneous datasets, we employed a domain-adversarial neural network (DANN) as a UDA framework to mitigate domain shifts [41]. The architecture of the UDA module is shown in Fig. S2B, and its description is provided in Supplementary Method 1. The UDA model was trained using labeled data from the source domain and unlabeled data from the external target domain. During training, the gradient reversal layer (GRL) in the UDA module multiplies the gradient by a negative scalar during backpropagation, forcing the feature extractor to learn domain-invariant representations. The network thus jointly optimizes 2 objectives as defined in Eq. (1): minimizing the classification loss on the source domain and maximizing the confusion of the domain classifier in identifying between the source and target domains.

$$\min_{F,C}\;\max_{D}L_{\mathrm{cls}}\left(F,\;C\right)-\lambda L_{\text{domain}}\left(F,\;D\right)$$ (1)

In Eq. (1), the feature extractor of DenseNet F and SVCI classifier C minimize the source domain SVCI classification loss, and the domain classifier D is trained to classify data domains. λ is domain-adversarial scheduling parameter.

During adversarial training, the adaptively weighted scheduling parameter λ encourages stable adversarial training during the early epochs and stronger domain adaptation at later stages. Scheduling parameter λ for GRL was dynamically increased according to Eqs. (2.1) and (2.2):

$$\lambda =\frac{2}{\left(1+\exp\left(-5\cdot p\right)\right)}-1$$ (2.1)

$$p=\frac{i+\text{epoch}\cdot N_{\text{iter}}}{N_{\text{epoch}}\cdot N_{\text{iter}}}$$ (2.2)

In Eq. (2.2), i is the current iteration index within an epoch, $N_{\text{iter}}$ is the total number of iterations per epoch, and $N_{\text{epoch}}$ is the total number of training epochs. The parameter *p* is the training progress, linearly changing from 0 to 1.

The domain classification loss and SVCI classification loss were optimized jointly using a weighted sum of losses in Eq. (3):

$$L_{\text{total}}=L_{{\mathrm{cls}}_{\text{source}}}+\alpha \left(L_{\text{domai}n_{\text{source}}}+L_{\text{domai}n_{\text{target}}}\right)$$ (3)

where $L_{{\mathrm{cls}}_{\text{source}}}$ is the SVCI classification loss on source domain, $L_{\text{domai}n_{\text{source}}}$ and $L_{\text{domai}n_{\text{target}}}$ are the losses for domain classification on source domain and target domain, and α was set at 0.1.

The model was optimized using the Adam optimizer with a learning rate of 0.00001 and weight decay of 0.001. Training was performed for 200 epochs with a batch size of 4. To ensure balanced domain alignment, one mini-batch was sampled from both the source and target domains in each iteration with identical batch sizes, and the number of iterations per epoch was determined by the smaller dataset. All classifier layers were trained using cross-entropy loss. The full training procedure is summarized in Supplementary Method 1.

Model convergence was evaluated by monitoring the stability of the training losses across the source and target domains. During training, the source domain classification loss showed a gradual increase, while the target-domain loss decreased, and both losses eventually approached similar values. The decreasing oscillation amplitude of the 2 losses over successive epochs indicated that the adversarial optimization between the feature extractor and the domain classifier had reached a steady state. This stabilization of both losses was regarded as the convergence criterion for training.

The DenseNet with UDA strategy contains 574,364 parameters and 560.77 MB GPU usage, and the training time for the UDA model was approximately 19.02 min. All experiments were conducted on a workstation equipped with an NVIDIA Quadro RTX 8000 GPU.

### Evaluation of DenseNet performance

The performance of the DenseNet model was measured using accuracy, recall, specificity, F1 score, and area under the receiver operating characteristic curve (AUC). The formulas for accuracy, recall, specificity, F1 score, and AUC are defined in Eqs. (4) to (8). Receiver operating characteristic (ROC) curves were used to assess discriminative performance. Calibration curves were applied to evaluate the agreement between predicted probabilities and observed outcomes. Decision curve analysis (DCA) was conducted to assess the potential clinical utility of the models across a range of decision thresholds. The constructed DenseNet model was then evaluated using these performance metrics on the internal and target-domain test sets.

$$\text{accuracy}=\frac{\mathrm{TP}+\mathrm{TN}}{\mathrm{TP}+\mathrm{TN}+\mathrm{FP}+\mathrm{FN}}$$ (4)

$$\text{recall=}\frac{\mathrm{TP}}{\mathrm{TP}+\mathrm{FN}}$$ (5)

$$\text{specificity}=\frac{\mathrm{TN}}{\mathrm{TN}+\mathrm{FP}}$$ (6)

$$F1\;\text{score}=\frac{2\times \mathrm{TP}}{2\times \mathrm{TP}+\mathrm{FP}+\mathrm{FN}}$$ (7)

$$\begin{array}{c} \mathrm{AUC}=\sum_{i=1}^{n-1}\left({\mathrm{FP}}_{i+1}-{\mathrm{FP}}_{i}\right)\cdot \frac{{\mathrm{TP}}_{i+1}+{\mathrm{TP}}_{i}}{2} \end{array}$$ (8)

In Eqs. (4) to (8), the true positive (TP) is the number of SVCI patients correctly identified as SVCI. False positive (FP) is the number of non-SVCI patients incorrectly identified as SVCI. False negative (FN) is the number of SVCI patients incorrectly identified as non-SVCI patients. True negative (TN) is the number of non-SVCI patients correctly identified as non-SVCI patients.

### Visualization of salient regions

Guided backpropagation approach was adapted to visualize salient WM regions contributing to model decision-making [45]. This method backpropagates only the positive gradient from the output layer to the input layer, effectively filtering out less relevant features and highlighting positively contributing structures. The backpropagation process is defined in Eq. (9).

$$R_{i}^{l}=1\left(f_{i}^{l}>0\right)\times 1\left(R_{i}^{l+1}>0\right)\times R_{i}^{l+1}$$ (9)

In Eq. (9), $f_{i}^{l}$ is the pre-activation value (input to ReLU, obtained during the forward pass) in l layer of the model and $R_{i}^{l+1}$ is the backpropagated gradient in l+1 layer. The term $1\left(f_{i}^{l}>0\right)$ ensures that only positive activations during the forward pass are allowed to propagate the gradient, and $1\left(R_{i}^{l+1}>0\right)$ further restricts the backpropagation to positive gradients only. $R_{i}^{l}$ is the guided gradient at the l layer.

The trained DenseNet model generated an individual-level salient map for each patient in internal test set. The map quantified voxel-wise contribution to the model decision. To identify population-consistent salient regions and reduce individual noise, we performed one-sample *t* tests across individual maps [the resulting *t*-statistic map was thresholded at *P* < 0.05, corrected using the Gaussian random field (GRF) method]. The resulting group-level salient maps were projected onto the Johns Hopkins University (JHU) WM atlas to identify which WM regions contain significant saliency voxels. This helped us to know group-level salient regions.

### Neuropsychological relevance evaluation of DenseNet

To evaluate whether the model’s predicted SVCI probability reflects the severity of cognitive impairment, we analyzed the association between the predicted probabilities from DenseNet and 6 neuropsychological scores (MMSE, MoCA, Immediate Recall, Delayed Recall, TMT-A, and TMT-B). Pearson or Spearman correlation analysis was used depending on the distribution of the variables.

To evaluate the neuropsychological relevance of the salient regions for the DenseNet model’s decision-making, a convolutional neural network (CNN)-based regression model was built to examine the association between the DTI data within salient regions and neuropsychological scales. The architecture of the regression model is described in Supplementary Method 2 and Fig. S2C. In the internal dataset, patients with both DTI image data and 6 neuropsychological scales (MMSE, MoCA, Immediate Recall, Delayed Recall, TMT-A, TMT-B) available were included in the analysis. We used 80% data for the regression model training and 20% data for testing (training parameters are described in Supplementary Method 3). Pearson or Spearman correlation analysis (depending on the normality of variables) was performed to examine associations between the regression model estimated neuropsychological scores and true neuropsychological scores. We also evaluated the neuropsychological relevance of the DTI data within non-salient and whole-brain WM regions (details in Supplementary Method 4)

### Mapping neuropsychological scores to brain images

To explore the neuroanatomical correlates of neuropsychological scales, we computed voxel-wise MI between FA-MD data combination and 6 neuropsychological scores (MMSE, MoCA, Immediate Recall, Delayed Recall, TMT-A, TMT-B). The MI quantifies the statistical dependency between DTI images and neuropsychological performance, and is defined as:

$$\begin{array}{c} \mathrm{MI}\left(X,Y\right)=\sum_{x\in X}\sum_{y\in Y}p\left(x,y\right)\text{log}\frac{p\left(x,y\right)}{p\left(x\right)p\left(y\right)} \end{array}$$ (10)

In Eq. (10), $p\left(x\right)$ and $p\left(y\right)$ are the marginal probability distributions of variable x and variable y, and $p\left(x,y\right)$ is their joint distribution. Regions with high MI values indicate WM voxels whose microstructural variation carries the greatest information about variation in cognitive performance. Microstructural damage in these regions is the most informative and most closely associated with neuropsychological performance within each cognitive domain.

For each voxel, MI computation was performed using 100 permutation tests to ensure that the MI reflects genuine cognition-related structural features rather than noise; statistical significance was assessed at *P* < 0.05, corrected using GRF. The resulting MI maps were projected onto the JHU WM atlas for anatomical interpretation and visualization.

To assess the structural specificity of each neuropsychological scale MI map, we computed pairwise similarity between 6 MI maps using 4 metrics: Dice coefficient, Jaccard index, SSIM, and JS divergence. The definitions are as follows:

$$\text{Dice}=\frac{2\left|x\cap y\right|}{\left|x\right|+\left|y\right|}$$ (11)

$$\text{Jaccard}=\frac{\left|x\cap y\right|}{\left|x\cup y\right|}$$ (12)

$$\text{SSIM}=\frac{\left(2\mu_{x}\mu_{y}+C_{1}\right)\left(2\sigma_{\mathrm{xy}}+C_{2}\right)}{\left(\mu_{x}^{2}+\mu_{y}^{2}+C_{1}\right)\left(\sigma_{x}^{2}+\sigma_{y}^{2}+C_{2}\right)}$$ (13)

$$\begin{array}{c} \mathrm{JS}=\frac{1}{2}\sum_{i}p\left(x_{i}\right)\text{log}\frac{p\left(x_{i}\right)}{\frac{1}{2}\left(p\left(x_{i}\right)+p\left(y_{i}\right)\right)}\\ +\frac{1}{2}\sum_{i}p\left(y_{i}\right)\text{log}\frac{p\left(y_{i}\right)}{\frac{1}{2}\left(p\left(x_{i}\right)+p\left(y_{i}\right)\right)} \end{array}$$ (14)

In Eqs. (11) to (14), x and y are binarized MI images, and $\left|x\right|$ and $\left|y\right|$ are the non-zero voxels in each map. ∩ represents the intersection regions, and ∪ represents the union regions. In Eq. (13), $C_{1}$ and $C_{2}$ are small constants, $\mu_{x}$ and $\mu_{y}$ are the mean values of 2 maps, $\sigma_{x}$ and $\sigma_{y}$ represent their variances, and $\sigma_{\mathrm{xy}}$ denotes the covariance between the 2 maps. In Eq. (14), $p\left(x\right)$ and $p\left(y\right)$ are the normalized probability distributions of MI map x and y.

The anatomical overlap between group-level DenseNet salient maps and each MI map was quantified. This allowed us to construct a WM-neuropsychological scale mapping matrix, identifying the dominant WM regions that structurally support each neuropsychological scale.

### Similarity-based cognitive risk stratification

To investigate whether individual-level WM saliency patterns reflected distinct cognitive impairment levels, we computed the SSIM between each patient’s DenseNet-generated salient weight map and each neuropsychological MI map. SSIM quantifies how well an individual patient’s structural vulnerability pattern matches the population-level WM–cognition relevance pattern, thus providing a mathematically grounded measure of structure–function similarity. The resulting similarity scores quantified how closely a patient’s model-generated salient weight map aligned with neuropsychologically specific anatomical relevance. High SSIM values indicate that the model’s identification for an individual relies strongly on WM regions where microstructural damage has the highest statistical dependence on neuropsychological performance at the population level. For SVCI patients, these neuropsychologically related regions tend to exhibit substantial microstructural alterations, and thus, the model naturally focuses on these regions to inform its decision. SSIM quantifies the extent to which the model’s decision-making process engages WM regions that are biologically relevant to cognitive impairment. Higher SSIM corresponds to greater involvement of neuropsychological-critical regions and, consequently, a higher cognitive impairment risk.

For each cognitive domain, patients were clustered using unsupervised *K*-means clustering based on their SSIM scores. The optimal number of clusters (*K*) was determined using the elbow method. The resulting clusters were interpreted as representing different levels of structural-neuropsychological similarity, and thus different degrees of cognitive impairment risk. Statistical comparisons of similarity scores across groups were conducted using the Kruskal–Wallis test, followed by Dunn’s test with Bonferroni correction. From the clinical perspective, domain-specific risk stratification may thus help clinicians anticipate which functions are most likely to deteriorate. Patients showing selective high-risk patterns (e.g., high SSIM score in executive function-related neuropsychological scale) may benefit from customized cognitive training or tailored lifestyle interventions rather than applying uniform interventions.

To evaluate the robustness of SSIM-based clustering across different MI map constructions, we performed a simple sensitivity analysis. For each neuropsychological scale, we constructed MI maps using a random 80% subsample of subjects and then calculated subsample SSIM values using the subsample MI map. The same *K*-means clustering procedure was applied to the subsample SSIM values. We evaluated robustness using cluster-wise mean SSIM, cluster change rate, and Spearman correlation between full-sample and subsample SSIM values.

### Statistical analysis

Comparisons of continuous variables were performed using the *t* test or the Mann–Whitney *U* test. Comparison of categorical variables was performed using the chi-square test. Comparisons of AUC were performed using the DeLong test. Correlation analyses were performed using Pearson or Spearman methods. Comparisons between groups were performed using the Kruskal–Wallis test and Dunn’s test with Bonferroni correction. For all reported test metrics, 95% confidence intervals (CIs) were estimated using appropriate resampling or exact methods. Specifically, binomial exact confidence intervals were computed for accuracy, sensitivity, specificity, and AUC, while 95% CIs for F1 score were obtained by 2000 bootstrap resampling. For each bootstrap iteration, F1 was recalculated, and the median and 2.5 to 97.5 percentile range were reported as the 95% CI.

## Results

### Characteristics of the cohorts

We recruited a cohort of 134 SVCI patients and 171 age-matched SIVD patients as an internal dataset for model development. In the internal dataset, we randomly assigned 107 SVCI and 137 SIVD patients as the training set (80%), and 27 SVCI and 34 SIVD patients as the internal test set (20%). An external cohort comprising 90 SVCI patients and 103 age-matched SIVD patients was used as a target-domain dataset. Among them, 45 SVCI and 53 SIVD patients were used as unlabeled target-domain data for UDA model fitting, while the remaining 45 SVCI and 50 SIVD patients were reserved as an independent target-domain test set for performance evaluation. The relatively smaller number of SVCI patients in the external dataset reflects the real-world prevalence in the community-based cohort, where SIVD cases are more common than SVCI. Detailed demographic data are presented in Table 1. For both cohorts, there were no significant differences between SVCI patients and SIVD patients without cognitive impairment in terms of age, body mass index, or diabetes history. In the internal cohort, the education, gender ratio, hypertension history, MMSE, MoCA, Immediate Recall, Delayed Recall, TMT-A, TMT-B, Digit Forward Span, and Digit Backward Span showed differences between the SVCI and SIVD groups. In external cohorts, MMSE and MoCA showed significant differences between the SVCI and SIVD groups.

**Table 1.** Demographic information of datasets. There were minor cases of missing values in the demographics, which were filled with the mean value. Comparisons of subjects were performed by the chi-square test for categorical variables. If continuous variables satisfied the normal distribution, comparisons of continuous variables were performed by the *t* test; otherwise, they were performed by the Mann–Whitney *U* test. ***P < 0.001.

Demographic variable	Internal cohort number/% or mean (SD, score range)		External cohort number/% or mean (SD, score range)	
	SVCI patients	SIVD patients without cognitive impairment	SVCI patients	SIVD patients without cognitive impairment
Age/years	66.1 (7.3, 50–79)	67.3 (6.8, 52–83)	67.7 (7, 51–82)	68.3 (5.2, 55–79)
Education /years***	10.6 (2.9, 6–17)	12.3 (2.9, 6–17)	10.8 (3.5, 6–18)	11.9 (2.3, 6–16)
BMI/(kg·m^-2^)	25 (2.7, 19.1–30.1)	24 (3.2, 16.0–31.2)	25 (2.9, 19.2–30.8)	24 (3.4, 17.2–31.3)
Gender (male), *n* /%***	81 (60.4)	48 (28.1)	41 (45.6)	40 (38.8)
Hypertension, *n* /%***	96 (71.6)	90 (52.6)	52 (57.8)	64 (62.1)
Diabetes history, *n* /%	31 (23.1)	39 (22.8)	21 (23.3)	26 (25.2)
MMSE***	26.8 (2.4, 18–30)	29.0 (1.0, 26–30)	27.3 (2.1, 18–30)	29.5 (0.9, 27–30)
MoCA***	21.9 (3.7, 13–29)	26.8 (1.7, 23–30)	22.4 (3.6, 14–28)	26.1 (0.9, 23–30)
Immediate Recall***	21.7 (6.5, 9–34)	29.6 (4.6, 16–44)	–	–
Delayed Recall***	6.7 (3.3, 0–14)	11.4 (2.8, 0–15)	–	–
Trail Making Test-A***	76.5 (37.5, 24–178.6)	48.4 (22.7, 24.4–120)	–	–
Trail Making Test-B***	151.9 (82.4, 46–300)	85.4 (48.7, 32–277)	–	–
Digit forward span***	7.3 (1.2, 5–10)	8.1 (1, 5–10)	–	–
Digit backward span***	4.3 (1.2, 2–8)	5.2 (1.3, 3–8)	–	–

BMI, body mass index; MMSE, Mini-Mental State Examination; MoCA, Montreal Cognitive Assessment

### Performance of DenseNet model to identify SVCI from SIVD

The classification performance of DenseNet on the internal test set is summarized in Table 2. The FA-MD-RD model achieved the best performance on the internal test set, with the highest accuracy, recall, specificity, F1 score, and highest AUC of 0.902 (95% CI, 0.798 to 0.963), 0.889 (95% CI, 0.708 to 0.976), 0.912 (95% CI, 0.763 to 0.981), 0.902 (95% CI, 0.778 to 0.964), and 0.962 (95% CI, 0.879 to 0.994). The FA-MD-AD-RD model also achieved the highest AUC of 0.962 (95% CI, 0.879 to 0.994). The FA-MD model also achieved the highest accuracy of 0.902 (95% CI, 0.798 to 0.963) and F1 score of 0.902 (95% CI, 0.780 to 0.964). The DeLong test indicated that the AUCs of the AD model and the RD model were significantly lower than the highest AUC (*P* = 4.83 × 10^−2^, *P* = 4.75 × 10^−2^, respectively). From the perspective of AUC, all models except the AD model and the RD model showed no significant differences in identifying SVCI. From the perspective of accuracy, the FA-MD-RD model and the FA-MD model are both the best-performing models on the internal test set. To assess the superiority of DenseNet, the performances of other deep learning models were also compared (see Supplementary Result 1). The performance results of the model without data augmentation are also supplemented in Table S2.

**Table 2.** Performance comparison of the DenseNet model with different DTI data combinations. Data in parentheses are 95% CIs. *: *P* value is significant (<0.05) when comparing AUC values with the best AUC in the same dataset using the DeLong test.

Internal test set						
Diffusion scalar image combinations	Accuracy	Recall	Specificity	F1	AUC	*z* value of DeLong test
FA	0.836 (0.719–0.918)	0.852 (0.663–0.958)	0.824 (0.655–0.932)	0.837 (0.691–0.921)	0.916 (0.817–0.972)	1.665
MD	0.869 (0.758–0.942)	0.815 (0.619–0.937)	0.912 (0.763–0.981)	0.868 (0.722–0.941)	0.951 (0.863–0.990)	0.941
AD	0.820 (0.700–0.906)	0.704 (0.498–0.862)	0.912 (0.763–0.981)	0.817 (0.625–0.889)	0.920 (0.822–0.974) *	1.975
RD	0.869 (0.758–0.942)	0.815 (0.619–0.937)	0.912 (0.763–0.981)	0.868 (0.714–0.938)	0.936 (0.842–0.983) *	1.982
FA-MD	0.902 (0.798–0.963)	0.889 (0.708–0.976)	0.912 (0.763–0.981)	0.902 (0.780–0.964)	0.951 (0.863–0.990)	0.765
MD-AD	0.852 (0.738–0.930)	0.778 (0.577–0.914)	0.912 (0.763–0.981)	0.851 (0.692–0.928)	0.936 (0.842–0.983)	1.623
AD-RD	0.820 (0.700–0.906)	0.704 (0.498–0.862)	0.912 (0.763–0.981)	0.817 (0.619–0.889)	0.948 (0.859–0.988)	1.479
FA-AD	0.885 (0.778–0.953)	0.852 (0.663–0.958)	0.912 (0.763–0.981)	0.885 (0.745–0.952)	0.944 (0.854–0.987)	1.050
MD-RD	0.852 (0.738–0.930)	0.741 (0.537–0.889)	0.941 (0.803–0.993)	0.850 (0.684–0.920)	0.954 (0.868–0.991)	0.715
FA-RD	0.836 (0.719–0.918)	1.000 (0.872–1.000)	0.706 (0.525–0.849)	0.835 (0.732–0.931)	0.954 (0.868–0.991)	0.807
FA-MD-AD	0.836 (0.719–0.918)	0.741 (0.537–0.889)	0.912 (0.763–0.981)	0.834 (0.651–0.906)	0.962 (0.879–0.994)	1.72 × 10^−14^
MD-AD-RD	0.852 (0.738–0.930)	0.741 (0.537–0.889)	0.941 (0.803–0.993)	0.850 (0.682–0.920)	0.953 (0.866–0.991)	0.745
FA-AD-RD	0.869 (0.758–0.942)	0.815 (0.619–0.937)	0.912 (0.763–0.981)	0.868 (0.714–0.938)	0.951 (0.863–0.990)	0.839
FA-MD-RD	0.902 (0.798–0.963)	0.889 (0.708–0.976)	0.912 (0.763–0.981)	0.902 (0.778–0.964)	0.962 (0.879–0.994)	0.000
FA-MD-AD-RD	0.869 (0.758–0.942)	0.815 (0.619–0.937)	0.912 (0.763–0.981)	0.868 (0.714–0.938)	0.962 (0.879–0.994)	1.798 × 10^−14^

FA, fractional anisotropy; MD, mean diffusivity; AD, axial diffusivity; RD, radial diffusivity

**Fig. 2.**
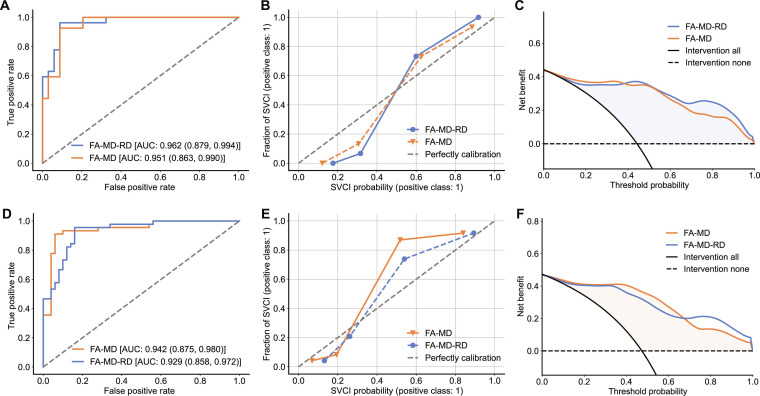
Performance of DenseNet. (A to C) Performance of FA-MD-RD model (blue) and FA-MD model (orange) on internal test set. (D to F) Performance of the same models after employing an unsupervised domain adaptation (UDA) strategy on the target-domain test set. (A and D) Receiver operating characteristic curves of the FA-MD-RD model (blue) and the FA-MD model (orange). The area under the curve (AUC) values are presented with 95% confidence intervals in parentheses. (B and E) Calibration curves comparing the model’s probabilities to observed SVCI incidence. Curves closer to the diagonal indicate better calibration. (C and F) Net benefit curves comparing the models’ decision-making utility. Higher net benefit indicates greater clinical utility. DTI, diffusion tensor images; FA, fractional anisotropy; MD, mean diffusivity; RD, radial diffusivity; FA-MD, DenseNet model derived from DTI combination FA and MD; FA-MD-RD, DenseNet model derived from DTI combination FA, MD, and RD.

We plotted ROC curves, calibration curves, and DCA curves for the FA-MD and FA-MD-RD models (as shown in Fig. 2A to C). The ROC curves in Fig. 2A show that the FA-MD-RD model outperforms the FA-MD model. The calibration curves in Fig. 2B show that the SVCI probabilities output from the models are close to the fraction of SVCI. The probability from the FA-MD-RD model is closer to the perfectly calibrated condition than the FA-MD model. The DCA curves in Fig. 2C show that the FA-MD-RD model yielded the greatest overall decision benefit. The FA-MD-RD model had a higher benefit than the FA-MD model when the threshold is over 0.38.

### Performance of UDA-DenseNet on the target-domain test set

We evaluated the performance of the constructed UDA-DenseNet model on a target-domain test set that was not involved in the UDA model fitting process. The best-performing models on the internal test set were further evaluated on the external cohort. The results are summarized in Table 3. The FA-MD DenseNet model attained the best performance, achieving an accuracy, recall, specificity, F1 score, and highest AUC of 0.926 (95% CI: 0.854 to 0.970), 0.911 (95% CI: 0.787 to 0.975), 0.940 (95% CI: 0.835 to 0.987), 0.926 (95% CI: 0.863 to 0.969), and 0.942 (95% CI: 0.875 to 0.980), respectively, on the target-domain test set. The FA-MD-RD model achieved the best performance on the internal test set but showed poorer performance on the target-domain test set. We conducted sensitivity analysis by varying the proportion of target-domain data used for UDA training (40%, 60%, 80%, and 100%). The data used for the sensitivity analysis did not overlap with the independent target-domain test set. Overall, The AUCs of the models remained relatively stable across different sampling proportions (Table S3), and no significant performance degradation was observed when fewer target-domain samples were used.

**Table 3.** Performance of the UDA-DenseNet model with different DTI data combinations. Data in parentheses are 95% CIs.

Target-domain test set—model with unsupervised domain adaptation						
Diffusion scalar image combinations	Accuracy	Recall	Specificity	F1	AUC	*z* value of DeLong test
FA-MD	0.926 (0.854–0.970)	0.911 (0.787–0.975)	0.940 (0.835–0.987)	0.926 (0.863–0.969)	0.942 (0.875–0.980)	0.000
FA-MD-AD	0.800 (0.705–0.875)	0.622 (0.465–0.762)	0.960 (0.863–0.995)	0.793 (0.703–0.872)	0.882 (0.800–0.939)	1.393
FA-MD-RD	0.853 (0.765– 0.917)	0.844 (0.705– 0.935)	0.860 (0.733–0.942)	0.853 (0.779–0.917)	0.929 (0.858–0.972)	0.416
FA-MD-AD-RD	0.832 (0.741–0.901)	0.756 (0.605–0.871)	0.900 (0.782–0.967)	0.830 (0.753–0.905)	0.864 (0.778–0.926)	1.605

FA, fractional anisotropy; MD, mean diffusivity; AD, axial diffusivity; RD, radial diffusivity

The performance of DenseNet without the UDA strategy on the target-domain test set was also evaluated (Table S4). Comparisons of AUCs between models with and without UDA using DeLong tests showed statistical significance for the FA-MD-AD-RD model (*P* = 0.006). The UDA strategy improved the accuracy, recall, specificity, and F1 score values of DenseNet models. Specifically, the FA-MD model without UDA achieved an accuracy of 0.884 (95% CI: 0.802 to 0.941) and AUC of 0.929 (95% CI: 0.857 to 0.972).

ROC curves, calibration curves, and DCA curves were plotted for the UDA-enhanced FA-MD model and the FA-MD-RD model on the target-domain test set (Fig. 2D to F). The ROC curves in Fig. 2D showed that the FA-MD model outperforms the FA-MD-RD model. The calibration curves in Fig. 2E showed that the FA-MD-RD model was closer to the perfectly calibrated condition. The DCA curves in Fig. 2F showed that the FA-MD model had a higher benefit than the FA-MD model when the threshold is lower than 0.67. The FA-MD model had higher benefits than the FA-MD-RD model. The ROC, calibration, and DCA curves for the FA-MD model and FA-MD-RD model without the UDA strategy on the target-domain test set are shown in Fig. S3. Without UDA, the FA-MD-RD model showed higher AUC than the FA-MD model. These results indicated that the UDA strategy improved FA-MD model performance on the target-domain test set compared with the model without UDA.

### Salient region visualization

**Fig. 3.**
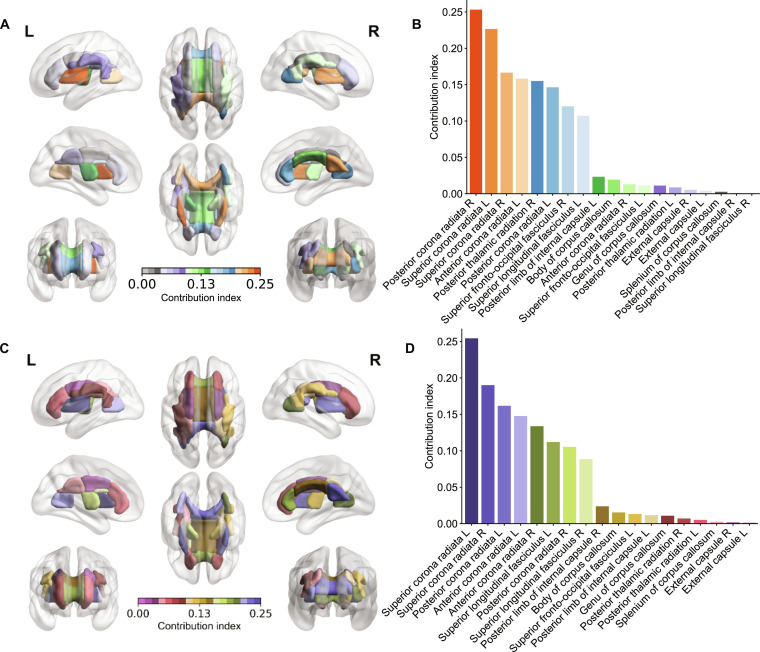
Visualization of salient brain regions. (A and B) Salient regions derived from DenseNet on the internal test set. (C and D) Salient regions derived from DenseNet employed UDA on the target-domain test set. (A and C) Spatial distribution of salient white matter brain regions. (B and D) Contribution weight of each salient white matter brain region. FA, fractional anisotropy; MD, mean diffusivity.

The FA-MD DenseNet model performed best on the internal and target-domain test sets. Guided backpropagation method was adopted to highlight the salient WM regions in the DTI data combination (FA, MD) for the model decision-making. There were 11 salient WM regions that contributed to the model decision: the superior, posterior, and anterior corona radiata; superior longitudinal fasciculus; posterior limb of internal capsule; body, genu, and splenium of corpus callosum; superior fronto-occipital fasciculus; posterior thalamic radiation; and external capsule (Fig. 3A and C).

Among the 11 influential WM regions, the corona radiata contributed the most to the model’s decision-making (Fig. 3B and D). Several salient regions are functionally associated with cognitive domains commonly impaired in SVCI. The superior longitudinal fasciculus and corona radiata are involved in attention and executive control, while the posterior thalamic radiation and splenium of the corpus callosum contribute to memory and visuospatial processing. The internal capsule and fronto-occipital fasciculus support cognitive-motor integration and long-range connectivity. This anatomical-functional correspondence suggests that the DenseNet model captures neurobiologically meaningful regions, aligning with the multidomain cognitive deficits observed in SVCI patients.

### Neuropsychological relevance of DenseNet

We evaluated the neuropsychological relevance of the DenseNet model from 2 perspectives: (a) the association between the predicted SVCI probability and 6 neuropsychological scores, and (b) the association between the model-focused salient regions and 6 neuropsychological scales. Forty-four SVCI and 60 SIVD patients from the internal training set with both DTI images and 6 neuropsychological assessments were included.

**Fig. 4.**
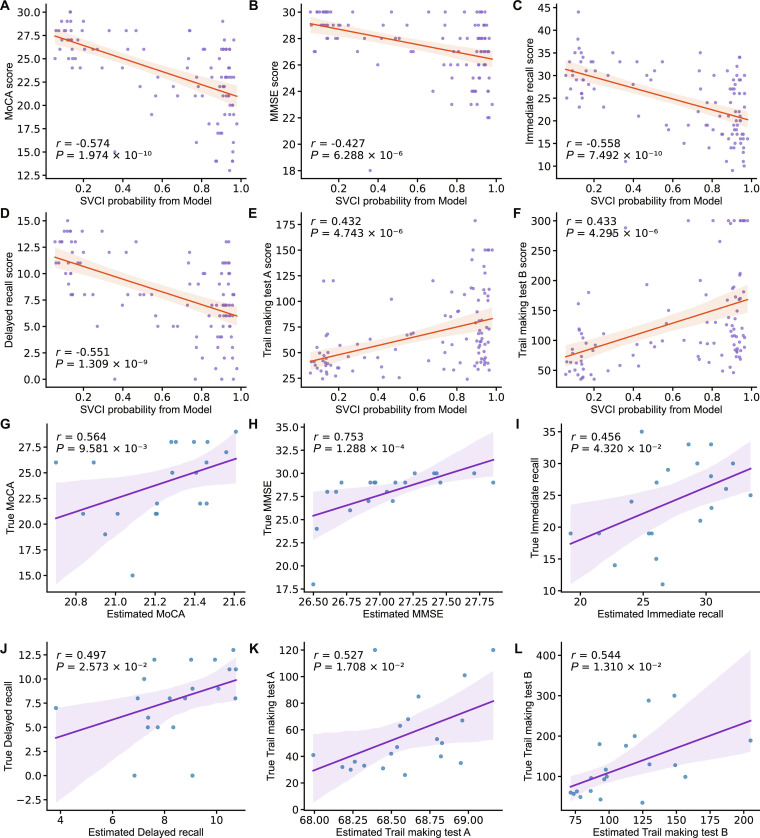
Neuropsychological relevance of DenseNet. (A to F) Correlation analysis between model output SVCI probabilities and 6 neuropsychological scales. (G to L) Correlation analysis between true neuropsychological scores and estimated neuropsychological scores using DTI data within DenseNet-generated salient regions. MoCA, Montreal Cognitive Assessment; MMSE, Mini-Mental State Examination; *r*, Pearson correlation coefficient. The solid line is the linear regression fitted line of the scattered points; the shaded part represents the 95% confidence interval.

We assessed the correlation between the model-predicted SVCI probabilities and neuropsychological scores using Pearson correlation analysis, after confirming normality (*P* < 0.05). Correlation results are illustrated in Fig. 4A to F. Six neuropsychological scales were significantly correlated with the DenseNet-derived SVCI probability. The global cognitive scale MoCA (Pearson: −0.574, *P* = 1.974 × 10^−10^) was the highest correlated with SVCI probabilities, followed by MMSE (Pearson: −0.427, *P* = 6.288 × 10^−6^). The memory functional scales Delayed Recall (Pearson: −0.551, *P* = 1.309 × 10^−9^) and Immediate Recall (Pearson: −0.558, *P* = 7.492 × 10^−10^), and the executive function-related scales TMT-A (Pearson: 0.432, *P* = 4.743 × 10^−6^) and TMT-B (Pearson: 0.433, *P* = 4.295 × 10^−6^) also correlated with the SVCI probabilities. The direction of correlation reflects the scoring nature of each neuropsychological scale: Higher MoCA, MMSE, Delayed Recall, and Immediate Recall scores indicate better cognition, hence negatively correlating with SVCI probability, whereas longer TMT-A/B completion times reflect worse executive function, showing positive correlations. These findings suggest that the model’s predicted probability reflects overall cognitive burden and domain-specific impairments.

We also assessed the neuropsychological relevance of the model-focused salient regions. We trained CNN-based regression models to estimate neuropsychological scores using DTI data combination (FA, MD) within salient WM regions (corona radiata, longitudinal fasciculus, internal capsule, corpus callosum, fronto-occipital fasciculus, thalamic radiation, and external capsule). Pearson correlation analysis results between estimated neuropsychological scores and true neuropsychological scores are shown in Fig. 4G to L. Six neuropsychological scales significantly correlated with DTI data within the salient WM regions. The strongest correlations were observed for the global cognitive scales MoCA (Pearson: 0.564, *P* = 9.581 × 10^−3^) and MMSE (Pearson: 0.753, *P* = 1.288 × 10^−4^). The memory functional scales Delayed Recall (Pearson: 0.497, *P* = 2.573 × 10^−2^) and Immediate Recall (Pearson: 0.456, *P* = 4.320 × 10^−2^), and the executive function-related scales TMT-A (Pearson: 0.527, *P* = 1.708 × 10^−2^) and TMT-B (Pearson: 0.544, *P* = 1.310 × 10^−2^) also correlated with the DTI data from salient regions. These results support the neuropsychological relevance of the model. Comparative results from non-salient regions and whole-brain regions are provided in Supplementary Result 2, Fig. S4.

### Mapping results of neuropsychological scores to brain images

Forty-four SVCI patients from the internal training set with both DTI images and 6 neuropsychological assessments were included. We computed voxel-wise MI between the DTI data combination (FA, MD) and 6 neuropsychological scores in SVCI patients. Permutation testing identified 6 statistically significant MI maps (*P* < 0.05, GRF corrected), each corresponding to a specific neuropsychological scale. The spatial distributions of these maps, visualized in the JHU WM atlas, are shown in Fig. S5.

To assess the structural specificity of each neuropsychological scale, we computed pairwise similarity metrics (Dice, Jaccard, SSIM, Jensen–Shannon (JS) divergence) across the 6 MI maps. The results are displayed as heatmaps in Fig. S6, revealing low intermap overlap (mean Dice: 0.057 ± 0.022, Jaccard: 0.030 ± 0.012, and SSIM: 0.294 ± 0.008). JS divergence score was 0.807 ± 0.010, revealing interscale structural variability.

**Fig. 5.**
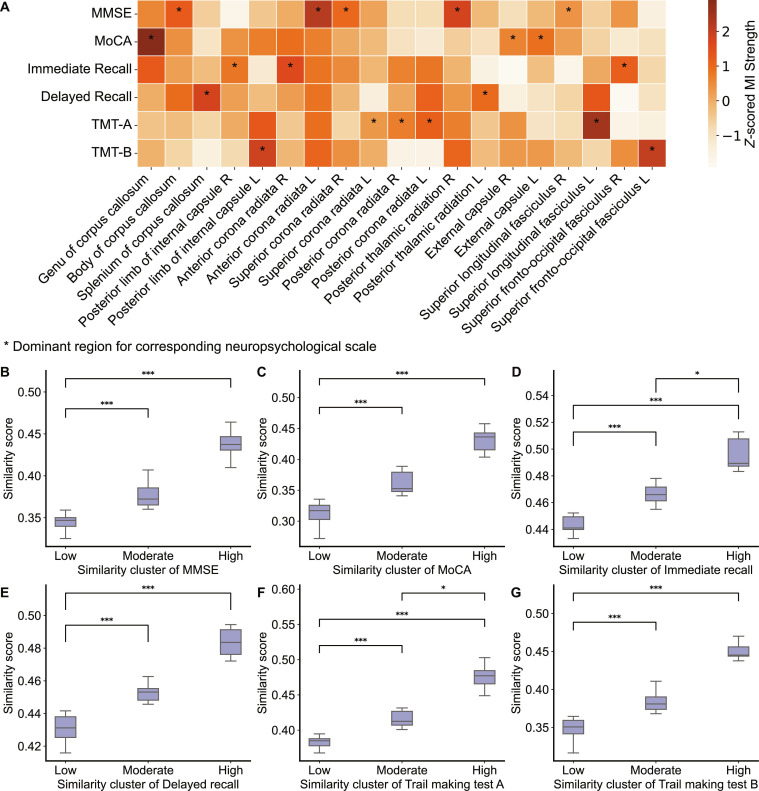
Imaging-based cognitive profiling. (A) White matter-neuropsychological relevance mapping matrix. The matrix displays the mutual information (MI) values of the salient regions identified by the DenseNet model in the MI maps of 6 neuropsychological scales. For each region, the neuropsychological scale with the highest *z*-scored MI value was considered its dominant associated domain. (B to G) Each subplot illustrates the clustering of SVCI patients based on structural similarity index measure (SSIM) scores between individual-level model-generated salient weight maps and domain-specific MI maps. For each scale, patients were grouped into 3 clusters (low, moderate, and high) using *K*-means clustering. (B) Clustering for MMSE. (C) Clustering for MoCA. (D) Clustering for Immediate Recall. (E) Clustering for Delayed Recall. (F) Clustering for Trail Making Test-A. (G) Clustering for Trail Making Test-B. Statistical differences between clusters were assessed using the Kruskal–Wallis test and Dunn’s test with Bonferroni correction. *P < 0.05; ***P < 0.001. MoCA, Montreal Cognitive Assessment; MMSE, Mini-Mental State Examination.

To identify WM regions associated with each neuropsychological scale, we overlapped the MI maps with DenseNet-derived group-level salient regions and extracted the dominant WM regions per scale (Fig. 5A). Damage in dominant regions may primarily contribute to deficits in that cognitive domain. MMSE was primarily associated with the body of the corpus callosum, left anterior corona radiata, right superior corona radiata, right posterior thalamic radiation, and right superior longitudinal fasciculus; MoCA with genu of corpus callosum and bilateral external capsule; Immediate Recall with the right posterior limb of internal capsule, right anterior corona radiata, and right superior fronto-occipital fasciculus; Delayed Recall with the splenium of corpus callosum and left posterior thalamic radiation; TMT-A with the left superior corona radiata, bilateral posterior corona radiata, and left superior longitudinal fasciculus; and TMT-B with the left posterior limb of internal capsule and left fronto-occipital fasciculus. These findings suggest that distinct neuroanatomical correlates underpin each neuropsychological scale in SVCI patients.

### SSIM-based cognitive risk stratification

We computed the SSIM between each SVCI patient’s DenseNet-generated salient weight map and the 6 domain-specific MI maps. For each neuropsychological scale, *K*-means clustering based on SSIM scores identified 3 subgroups (low, moderate, and high similarity), as determined by the elbow method (Fig. S7).

Dunn’s test with Bonferroni correction confirmed significant differences in SSIM scores between subgroups within each neuropsychological scale. Clustering results are shown in Fig. 5B to G. Specifically, for MMSE, significant differences were observed between low versus moderate (*P* = 1.739 × 10^−5^) and low versus high (*P* = 2.976 × 10^−7^); MoCA showed significant differences between low versus moderate (*P* = 3.634 × 10^−5^) and low versus high (*P* = 2.269 × 10^−5^); Immediate Recall showed significant differences across all pairs: low versus moderate (*P* = 1.019 × 10^−4^), low versus high (*P* = 2.252 × 10^−8^), and moderate versus high (*P* = 1.977 × 10^−2^). For Delayed Recall, significant differences were found for low versus moderate (*P* = 6.755 × 10^−5^) and low versus high (*P* = 4.464 × 10^−6^). TMT-A showed significance across all pairs: low versus moderate (*P* = 5.097 × 10^−5^), low versus high (*P* = 5.983 × 10^−8^), and moderate versus high (*P* = 2.786 × 10^−2^). TMT-B exhibited significant differences for low versus moderate (*P* = 6.455 × 10^−6^) and low versus high (*P* = 2.809 × 10^−6^).

We performed a sensitivity analysis using 80% subsamples of subjects. Table S8 shows cluster-wise mean SSIM values, cluster label change rate, and Spearman correlation of SSIM values for the full sample and subsample MI maps. The cluster change rate was consistently low across 6 neuropsychological scales (range, 0.000 to 0.318; median, 0.103). The Spearman correlations of cluster-wise SSIM values between full-sample and subsample maps remained high across all scales, with *r* values ranging from 0.880 to 0.996 (all *P* < 0.001). The results indicate that the SSIM-based clusters are largely stable across different MI map constructions, supporting the robustness of our main clustering results.

Patients in higher SSIM clusters (higher anatomical concordance with MI maps) exhibited worse cognitive performance. These results suggest that the WM salient pattern derived from the DenseNet model associated with each neuropsychological scale may reflect varying degrees of cognitive impairment.

## Discussion and Conclusion

In the present study, we proposed a DTI-based deep learning framework for SVCI. First, we constructed a DTI-based model that identifies SVCI from SIVD. Second, we introduced an imaging-driven method for multidomain cognitive risk profiling in SVCI patients, supporting individualized cognitive assessment.

Our DenseNet model achieved strong performance across both internal and target-domain test sets, and generated individual-level SVCI probabilities that were significantly correlated with neuropsychological scores. This enables DenseNet to accurately identify SVCI from SIVD patients. SVCI probability output offers clinicians an interpretable, continuous measure of cognitive impairment severity and may serve as a complementary reference in diagnostic decision-making. Model-generated salient WM regions—predominantly located in association and projection fiber tracts—showed significant associations with neuropsychological scores, demonstrating that the data-driven model is also neuropsychologically meaningful. Beyond classification, we performed individualized cognitive profiling based on images. First, we mapped the neuroanatomical correlates of 6 neuropsychological scales by computing MI between DTI and neuropsychological scores. The resulting MI maps revealed domain-specific structural vulnerability patterns, confirming the heterogeneity of cognitive impairment in SVCI. To translate these maps into individual-level insights, we further calculated the SSIM between each patient’s salient weight map and the MI maps. Finally, unsupervised clustering based on SSIM scores identified distinct cognitive risk subgroups, with higher SSIM scores associated with significantly poorer cognitive performance. These results demonstrated the feasibility of imaging-driven personalized cognitive risk stratification.

Compared to existing SVCI studies, our study presented multiple methodological and translational advantages (Table S6). Prior machine learning studies in SVCI relied on handcrafted radiomic or regional features, which are sensitive to preprocessing pipelines and may not generalize across datasets [32,33,46–48]. Our DenseNet model extracted hierarchical representations directly from raw DTI volumes with minimal manual processing. Model performance degradation across imaging domains remains largely unaddressed in prior studies [38–40]. We implemented the UDA strategy to improve the model’s performance on external data. Our use of DTI enhanced sensitivity to subtle WM microstructural alterations compared to structural MRI. Existing studies relied on T1-weighted or T2-FLAIR images for SVCI identification [38–40]. Such modalities often fail to capture early or diffuse WM pathology. DTI-derived metrics such as FA and MD reflect axonal integrity and tissue organization, offering a more pathophysiological basis for SVCI identification. This may be advantageous for identifying vascular-related cognitive impairment. Beyond model performance, our study integrated neuropsychological relevance analyses. None of the prior studies summarized in Table S6 examined relationships between model-derived representations and cognitive domains. By combining DenseNet with SSIM-based cognitive profiling, we demonstrated that imaging-driven model outputs are meaningfully associated with domain-specific cognitive impairments. Our study benefited from a well-maintained clinical cohort that includes paired DTI scans and comprehensive neuropsychological assessments covering 6 cognitive domains. We included an independent external test cohort with DTI data, enabling preliminary evaluation of cross-site performance. Importantly, the cohort is prospectively maintained with ongoing patient recruitment and follow-up, providing opportunities for future longitudinal analyses.

The salient WM regions identified by our DenseNet model, including projection, association, and commissural fibers, are consistent with known microstructural alterations in SVCI. Reduced FA and increased MD in the corona radiata, posterior limb of the internal capsule, external capsule, and corpus callosum have been recognized as hallmarks of subcortical small vessel disease [49–51]. Correlation analyses further confirmed that these regions are associated with performance across multiple cognitive domains, including memory, executive function, and attention. These findings support the notion that distributed WM damage contributes to the multidomain cognitive deficits observed in SVCI [51–56]. Model-generated salient regions reflect imaging patterns that are informative for both disease identification and neuropsychological relevance, providing an interpretable representation of the features contributing to the model’s decision-making. Deep learning-identified WM regions can serve not only as diagnostic markers but also as functional proxies for cognitive impairment, even in the absence of direct behavioral labels. This connects diagnostic modeling with individual-level cognitive profiling and enables the investigation of representation-level associations between imaging patterns and cognitive performance. The model provided voxel-wise saliency maps for each subject, allowing identification of patient-specific WM regions that contribute most strongly to cognitive impairment classification. This enables a personalized structural “risk signature”, which may complement conventional radiological interpretabilities, especially when early WM disruption is subtle and visually indistinguishable.

Building on the neuropsychological relevance of model-generated salient maps, we explored whether an individual-level salient weight map could reflect domain-specific cognitive impairment. Cognitive impairment in SVCI is heterogeneous, affecting different cognitive domains across individuals [53]. Clustering methods have been applied to uncover latent cognitive risk profiles [32,57]. However, few imaging-based approaches have been proposed to characterize SVCI’s cognitive impairment heterogeneity at the individual level. By computing SSIM between each patient’s salient weight map and domain-specific MI maps, we stratified SVCI patients into distinct cognitive risk subgroups. Patients with higher similarity exhibited worse cognitive performance. These findings suggest that distinct WM patterns underlie cognitive risk heterogeneity in SVCI, and that domain-specific saliency-MI similarity may serve as an imaging surrogate for cognitive profiles. This extends beyond global classification and highlights the potential of deep learning-based interpretability to capture fine-grained cognitive phenotypes for individualized assessment.

The present study has several limitations that should be acknowledged. First, the cohort size, while substantial for a single-disease neuroimaging study and sufficient as a proof of concept, remains relatively modest for deep learning training. This may limit the model’s capacity to capture the full spectrum of disease heterogeneity and increase the potential for overfitting, despite our use of mitigation strategies such as data augmentation, domain adaptation, and rigorous cross-validation. Second, the generalizability of our framework requires further validation. Although we successfully employed domain adaptation and external testing from a different hospital, the model’s performance in highly heterogeneous clinical environments, across diverse ethnic populations, and with drastically different scanner protocols needs to be confirmed through large-scale, multi-center international studies. Although all imaging data in this study were acquired using the same scanner manufacturer, and a unified preprocessing pipeline (including normalization to MNI space) to minimize site-related variability, we did not perform additional post-ComBat cross-site normalization. The residual subtle site effects may not be fully excluded. Third, the cross-sectional analyses presented here utilize the baseline data from our VIVA cohort. Therefore, the model identifies associations between WM integrity and cognitive status at a single time point. This inherently limits the prognostic value of the model in predicting individual cognitive decline over time. Luckily, our VIVA cohort is undergoing annual follow-up assessments. The availability of longitudinal data will allow us to directly address this limitation. Furthermore, there are methodological constraints. The MI maps used for similarity estimation were derived from a limited sample, which may introduce bias or instability. While the SSIM-based cognitive stratification yielded clinically meaningful subgroups, these clusters lack validation against long-term cognitive outcomes. SSIM primarily compares spatial distribution patterns and does not directly measure causal or mechanistic structure–function relationships. Future work could integrate multimodal functional imaging data (e.g., functional MRI connectivity or metabolic imaging) to strengthen the inference of structural–functional coupling. Finally, our cognitive profiling is based on neuropsychological scales and their WM correlates; future work would benefit from incorporating additional blood biomarkers to achieve a more comprehensive pathophysiological understanding.

In conclusion, the present study developed and validated a DTI-based DenseNet model to address key challenges in identifying SVCI and SIVD. Our DenseNet model not only achieved high diagnostic accuracy for SVCI across 2 clinical centers. Besides, the workflow required only DTI sequences in clinical MRI protocols, making it applicable for integration into standard clinical practice. Beyond diagnosis, we introduced a novel paradigm for multi-domain cognitive risk profiling. Our cognitive profiling approach provided an imaging-driven tool to assess the risk of impairment in specific cognitive functions, paving the way for personalized intervention strategies tailored to a unique pattern of WM injury. Future work should aim to expand the dataset, incorporate multi-modal imaging, and integrate longitudinal follow-up of cognitive trajectories. Such advances would further enhance the clinical utility and predictive precision of the framework, accelerating its translation into routine neurocognitive care.

## Data Availability

All relevant data are within the manuscript and its additional files. The datasets in the current study are available from the corresponding authors on reasonable request.
