# Supplementary material for: Deep Learning for Classifying and Cognitive Profiling of Subcortical Vascular Cognitive Impairment
Source: Cyborg Bionic Syst. 2026 May 13;7:0561. doi: 10.34133/cbsystems.0561 (PMC13168761; doi:10.34133/cbsystems.0561)
Supplement: Supplementary 1 — Supplementary Text Figs. S1 to S7 Tables S1 to S8 [file cbsystems.0561.f1.docx]

SUPPLEMENTARY MATERIALS

**SUPPLEMENTARY METHODS**

1. **UDA model structure and training procedure**

To enhance the performance of the DenseNet model on unseen target-domain data, a UDA module based on the DANN framework was incorporated[41]. A gradient reversal layer and a domain classifier were attached to the DenseNet feature extractor to encourage learning of domain-invariant representations. The GRL was inserted between the feature extractor and the domain classifier. The domain classifier was implemented as a three-layer fully connected network (input dimension equal to the feature dimension of the feature extractor). Specifically, it consisted of three linear layers with sizes 256 → 128 → 2, each followed by batch normalization, ReLU activation, and dropout (rate = 0.5).

The pseudo code of the UDA training procedure is described as follows.

|  | **Algorithm 1 Unsupervised Domain Adaptation Training for DenseNet** |
| --- | --- |
| 1: | Input: Source dataset $D_{s}=\left[ \left( x_{s},y_{s} \right) \right]$; Target dataset $D_{t}=\left[ x_{t} \right]$;  Model: feature extractor $F$; label classifier $C$; domain classifier $D$;  gradient reversal layer $GRL$;  Parameters: cross entropy loss function $CE$; loss weight $\alpha$; learning rate $\eta$; epochs $N$;  batch size $b$ |
| 2: | Initialize model parameters (DenseNet pre-trained on $D_{S}$) |
| 3: | **for** epoch = 1 to $N$ **do** |
| 4: | **for** each mini-batch $\left( x_{s},y_{s} \right)\in D_{s}$ and $\left( X_{t} \right)\in D_{t}$ **do** |
| 5: | $p$ = (current_iteration + epoch * total_iterations) / ($N$ * total_iterations) |
| 6: | $\lambda=2/(1+e^{-5*p}))-1$ |
| 7: | $f_{s}=F(x_{s})$; |
| 8: | $f_{t}=F(x_{t})$ |
| 9: | $\hat{y}_{s}, \hat{d}_{s}=C\left( f_{s} \right), D(GRL(f_{s}, \lambda))$ |
| 10: | $\hat{d}_{t}=D(GRL(f_{t},\lambda))$ |
| 11: | $L_{cls}=CE(\hat{y}_{s}, y_{s})$ |
| 12: | $L_{domain}=CE\left( \hat{d}_{s}, domain_{label}=0 \right)+CE(\hat{d}_{t}, domain_{label}=1)$ |
| 13: | $L_{total}=L_{cls}+ \alpha*L_{domain}$ |
| 14: | $Update\left( F, C, D \right)\leftarrow Adam(\nabla L_{total}, \eta)$ |
| 15: | **end for** |
| 16: | **end for** |

**2. Regression model architecture**

To evaluate the association between DTI data within DenseNet-generated salient regions and neuropsychological scales, we constructed a 3D regression model based on DenseNet architecture to estimate neuropsychological scores. The architecture of the regression model is described in Fig. S2C. The model takes 3D dual-channel image data as input (input shape 2×91×109×91), processes it through a series of residual convolutional modules for feature extraction, and utilizes a fully connected layer to output the estimated neuropsychological scores. The network consists of an initial convolutional layer, followed by four dense blocks interleaved with three transition layers. Each dense block is composed of one densely connected 3D convolutional layer with a growth rate of 32. Global average pooling is applied before a fully connected linear layer to generate the final prediction. A dropout rate of 0.5 is applied before the final layer.

**3. Regression model training**

The model was trained using mean squared error loss to regress the target neuropsychological scale. The model was trained with a batch size of 4 and a learning rate of 0.0001 for 30 epochs. The Adam optimizer was used to optimize the model’s parameters with weight decay set at 0.001.

**4. Evaluate the neuropsychological relevance of the non-salient and whole-brain white matter for model decision-making.**

We additionally analyzed the neuropsychological relevance of non-salient regions (regions not illustrated in Fig. 3A) for the DenseNet model’s decision-making and whole-brain DTI data. In this analysis, we trained CNN-based regression models using DTI data from the non-salient regions and whole-brain white matter. The output of the CNN regression model was the estimated neuropsychological scores. Pearson or Spearman correlation analysis (depending on the normality of the variables) was then performed to assess the associations between the estimated neuropsychological scores and the true neuropsychological scores.

**SUPPLEMENTARY RESULTS**

**1. Comparison of deep learning models**

To assess the superiority of the DenseNet model applied in this study, we also used VGG11, ResNet18, and Transformer models to identify SVCI. These models were selected based on their prior use in deep learning studies for SVCI classification, suggesting they are potentially suitable for this task[38-40]. We trained these models using the same DTI data as used for DenseNet, and compared their performance in identifying SVCI with that of the DenseNet model developed in this study.

Performances of deep learning models are illustrated in Table S5. The DTI data combination (FA, MD) and (FA, MD, RD) were the two best-performing DTI data combinations for DenseNet development, so they were selected for comparison. The VGG11, ResNet18, and transformer models were then trained using these data combinations. The transformer model did not converge during the training phase, and therefore, its test performance is not listed in the table. The AUC values of the FA-MD-RD ResNet18 model were significantly lower than the greatest AUC achieved by FA-MD-RD DenseNet. VGG11 and ResNet18 models did not achieve higher accuracy than DenseNet. And the model performance in previous studies is summarized in Table S6. The number of model parameters and training epochs used for comparison are also summarized in Table S7.

**2. Neuropsychological relevance of DTI data within the non-salient of DenseNet and whole-brain regions.**

DTI data combination (FA, MD) within non-salient white matter regions (regions not in the salient regions illustrated in Fig. 3A) were significantly correlated with MoCA (Pearson:0.595, *p* = 5.626×10^-3^), Immediate Recall (Pearson: 0.492, *p* = 2.740×10^-2^), respectively (Fig. S4A-S4F). The whole brain regions of DTI data combination (FA, MD) were correlated with MoCA (Pearson: 0.575, *p* = 7.932×10^-3^), Immediate Recall (Pearson: 0.526, *p* = 1.710×10^-2^), and Trail Making Test-B (Pearson: 0.489, *p* = 2.877×10^-2^) (Fig. S4G-S4L).

**Fig S1.** Inclusion and exclusion process for cohort data used in the present study. VIVA, vascular impairment and vascular aging. MRI, magnetic resonance imaging; SVCI, subcortical vascular cognitive impairment; SIVD, subcortical ischemic vascular disease; AD, Alzheimer’s disease.


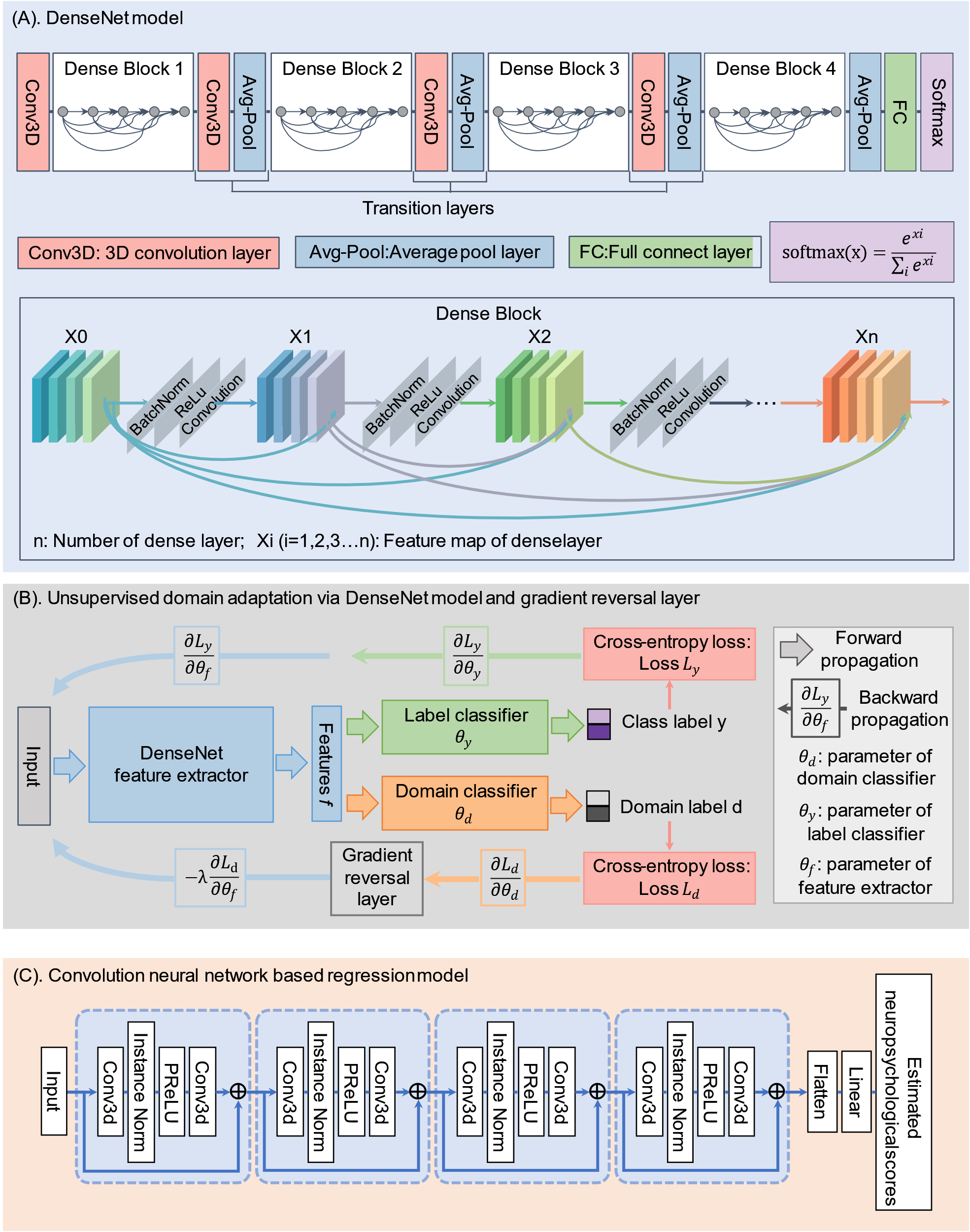


**Fig S2.** The network structures of DenseNet, the unsupervised domain adaptation network, and the regression model. (A) The DenseNet for SVCI diagnosis. (B) Structure of the unsupervised domain adaptation network. (C) Structure of the regression model for neuropsychological relevance evaluation.


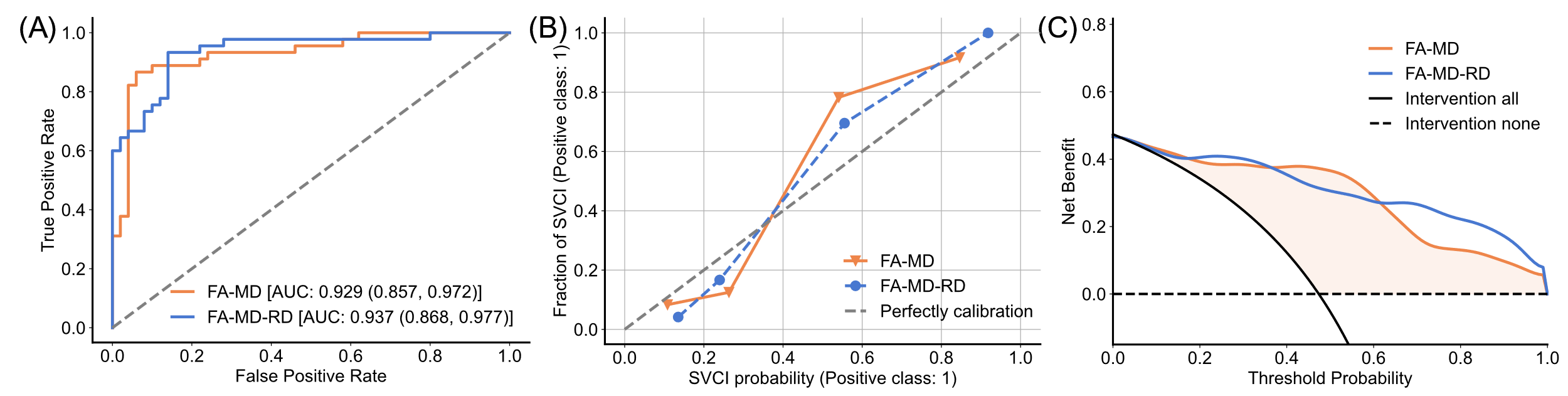


**Fig S3.** Performance of DenseNet without UDA on target-domain test set. Results are shown for the FA-MD-RD model (blue) and the FA-MD model (orange). (A) Receiver operating characteristic curves with the area under the curve (AUC) values and 95% confidence intervals in parentheses. (B) Calibration curves comparing the model’s probabilities to observed SVCI incidence. (C) Net benefit curves comparing the models' decision-making utility. UDA, unsupervised domain adaptation; FA, fractional anisotropy; MD, mean diffusivity; RD, radial diffusivity. FA-MD, DenseNet model derived from DTI combination FA and MD. FA-MD-RD, DenseNet model derived from DTI combination FA, MD, and RD.


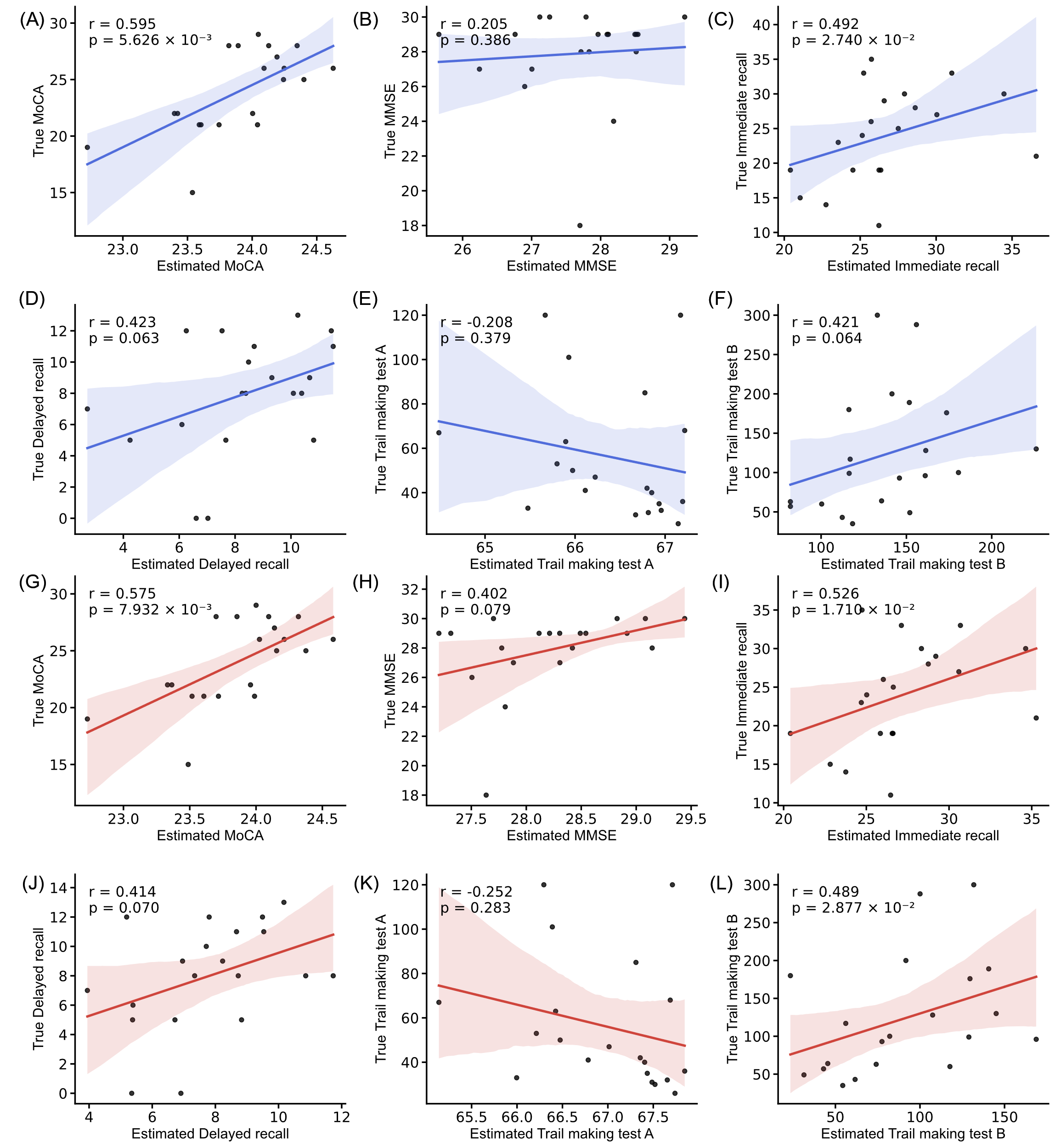


**Fig S4.** Neuropsychological relevance of DTI data within non-salient regions of DenseNet and whole brain. (A–F) Correlation results using non-salient white matter (WM) regions, for MoCA, MMSE, Immediate Recall, Delayed Recall, Trail Making Test-A, and Trail Making Test-B, respectively. (G–L) Correlation results using whole-brain WM regions, for the same six scales in the same order. r, Pearson Correlation coefficient; The solid line is the regression fitted line of the scattered points; the shaded part represents the 95% confidence interval.


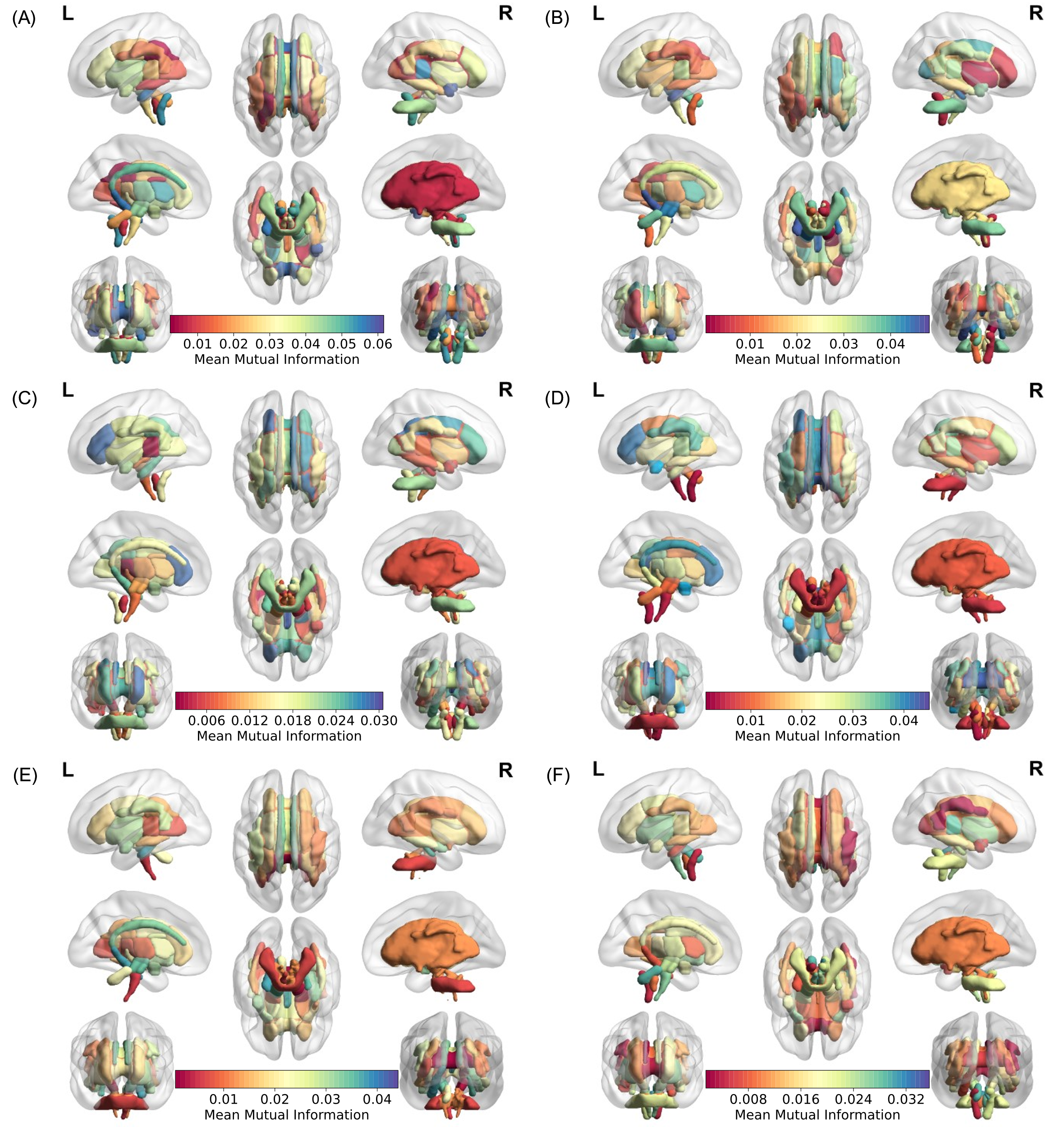


**Fig S5.** Spatial distribution visualization of MI maps. (A) MI maps of MoCA. (B) MI maps of MMSE. (C) MI maps of Immediate Recall. (D) MI maps of Delayed Recall. (E) MI maps of Trail Making Test-A. (F) MI maps of Trail Making Test-B.


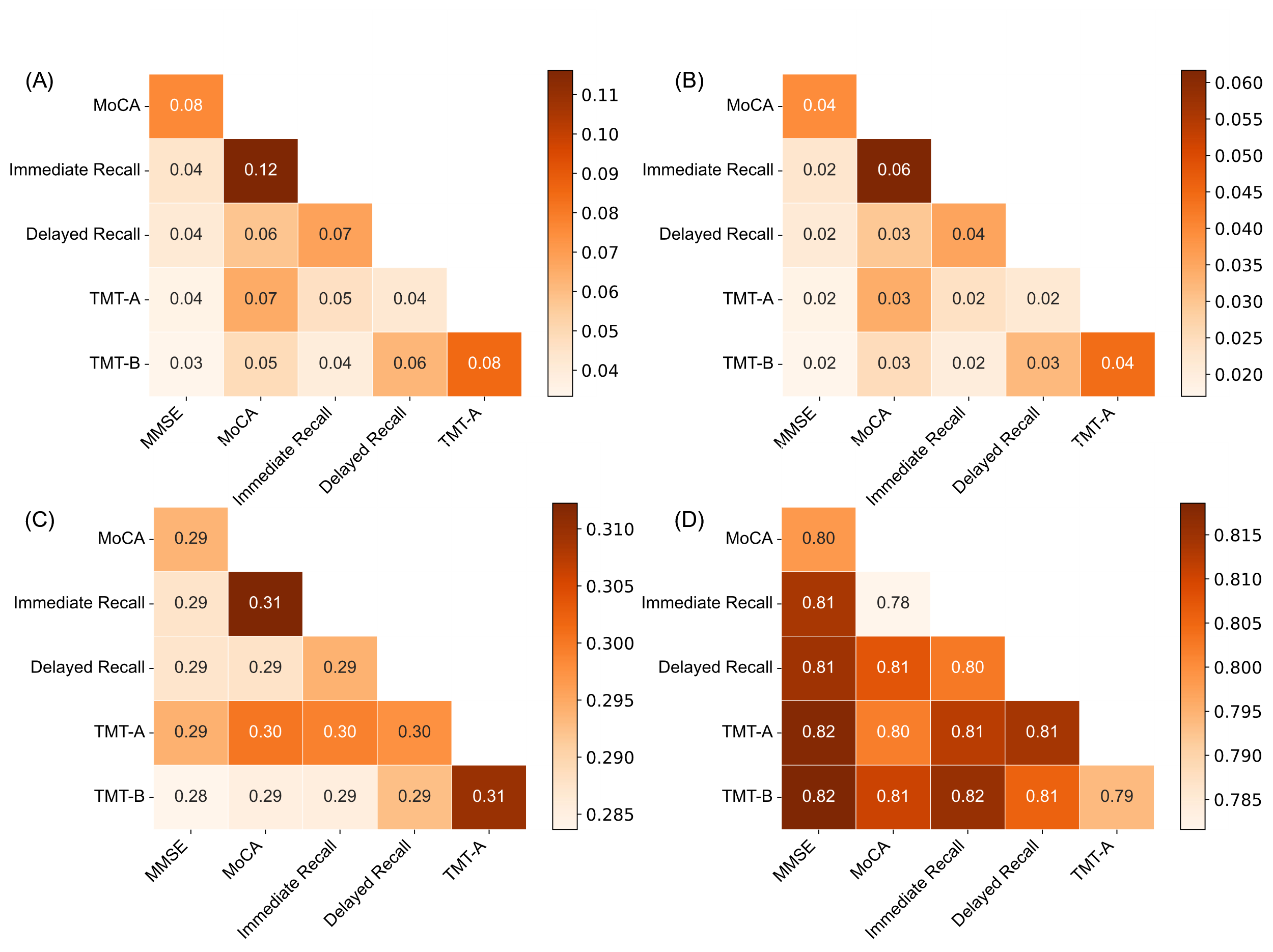


**Fig S6.** Structural specificity of six mutual information maps. (A) Pairwise Dice coefficient. (B) Pairwise Jaccard index. (C) Pairwise Structural similarity index. (D) Pairwise Jensen-Shannon divergence.


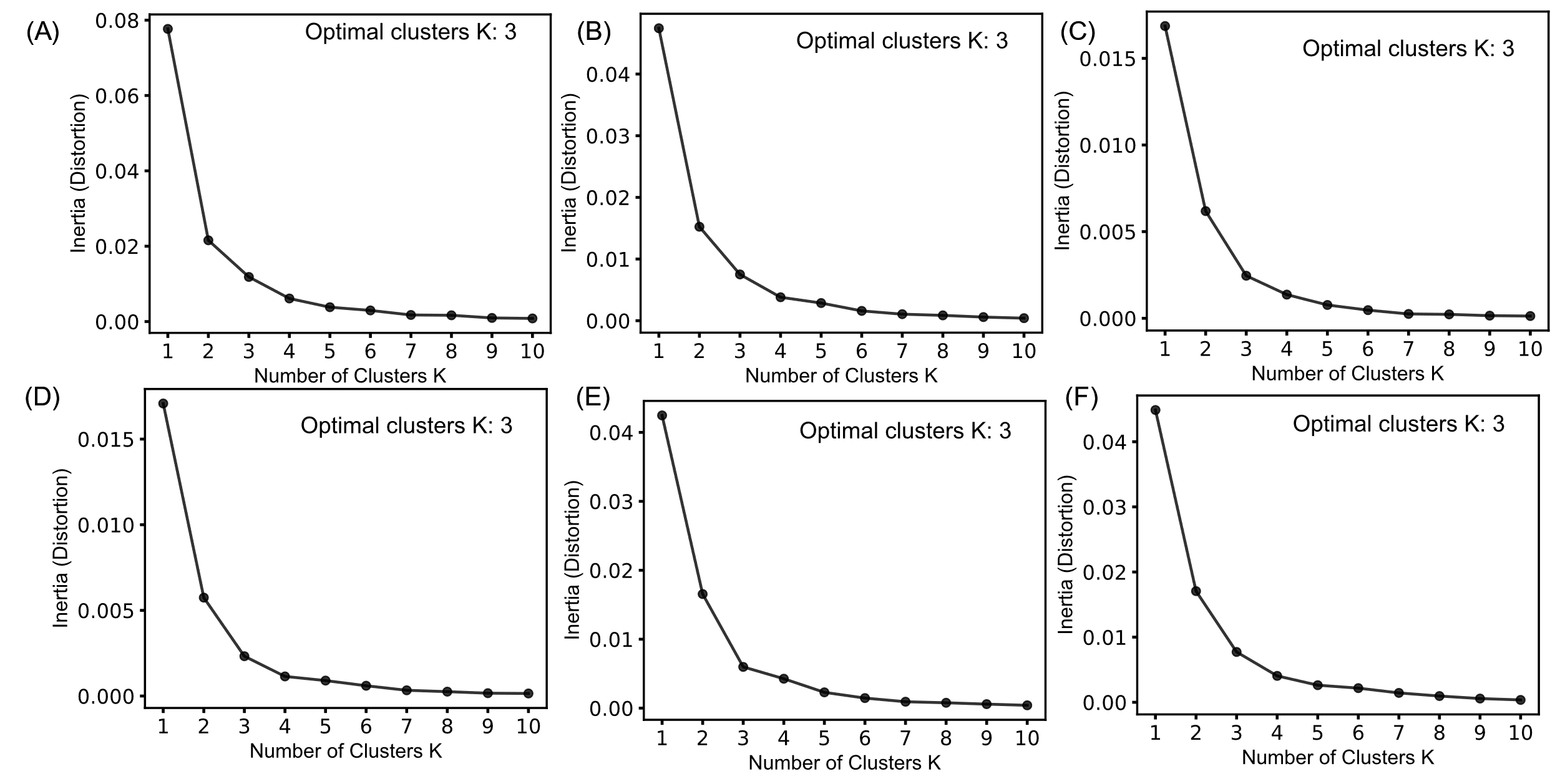


**Fig S7.** Determination of optimal number of clusters using the elbow method. Elbow plots for K-means clustering were generated based on structural similarity index measure (SSIM) scores between model-derived saliency maps and MI maps for each neuropsychological scale. The x-axis indicates the number of clusters (K), and the y-axis shows the within-cluster sum of squares (distortion score). The point at which the curve begins to flatten (elbow point) was used to determine the optimal K. (A) MoCA; (B) MMSE; (C) Immediate Recall; (D) Delayed Recall; (E) Trail Making Test-A; (F) Trail Making Test-B.

**Table S1.** Data augmentation summary

|  | **Augmentation** | **Parameter (range)** | **Applied probability** |
| --- | --- | --- | --- |
| Apply one of | Random flip | axis = 1 | 0.20 |
|  | Random zoom | zoom$\in$[0.9,1.1] | 0.20 |
| Apply one of | Intensity scale | zoom$\in$[0.9,1.1] | 0.30 |
|  | Intensity shift | offset ± 0.1 | 0.30 |
|  | Intensity normalization | channel-wise | 1.00 |

**Table S2.** Performance of DenseNet model on internal test set without data augmentation

| **Internal test set – model without data augmentation** | | | | | | |
| --- | --- | --- | --- | --- | --- | --- |
| Diffusion scalar  image combinations | Accuracy | Recall | Specificity | F1 | AUC | z value of  DeLong test |
| FA | 0.836  (0.719,0.918) | 0.852  (0.663,0.958) | 0.824  (0.655,0.932) | 0.837  (0.738,0.918) | 0.917  (0.818,0.972) | 1.814 |
| MD | 0.852  (0.738,0.930) | 0.852  (0.663,0.958) | 0.853  (0.689,0.950) | 0.853  (0.689,0.950) | 0.932  (0.838,0.981) | 1.599 |
| AD | 0.820  (0.700,0.906) | 0.704  (0.498,0.862) | 0.912  (0.763,0.981) | 0.817  (0.706,0.916) | 0.936  (0.842,0.983) | 1.485 |
| RD | 0.852  (0.738,0.930) | 0.815  (0.619,0.937) | 0.882  (0.725,0.967) | 0.852  (0.755,0.934) | 0.948  (0.859,0.988) | 1.289 |
| FA-MD | 0.852  (0.738,0.930) | 0.815  (0.619,0.937) | 0.882  (0.725,0.967) | 0.852  (0.756,0.934) | 0.942  (0.851,0.986) | 1.071 |
| MD-AD | 0.836  (0.719,0.918) | 0.778  (0.577,0.914) | 0.882  (0.725,0.967) | 0.835  (0.735,0.918s) | 0.937  (0.844,0.983) | 1.515 |
| AD-RD | 0.885  (0.778,0.953) | 0.852  (0.663,0.958) | 0.912  (0.763,0.981) | 0.885  (0.802,0.952) | 0.947  (0.857,0.988) | 1.242 |
| FA-AD | 0.852  (0.738,0.930) | 0.852  (0.663,0.958) | 0.853  (0.689,0.950) | 0.853  (0.689,0.950) | 0.940  (0.848,0.985) | 1.370 |
| MD-RD | 0.885  (0.778,0.953) | 0.926  (0.757,0.991) | 0.853  (0.689,0950) | 0.886  (0.804,0.952) | 0.958  (0.872,0.993) | 0.524 |
| FA-RD | 0.869  (0.758,0.942) | 0.852  (0.663,0.958) | 0.882  (0.725,0.967) | 0.869  (0.772,0.950) | 0.959  (0.874,0.993) | 0.431 |
| FA-MD-AD | 0.869  (0.758,0.942) | 0.926  (0.757,0.991) | 0.824  (0.655,0.932) | 0.869  (0.774,0.951) | 0.960  (0.876,0.993) | 0.486 |
| MD-AD-RD | 0.836  (0.719,0.918) | 0.815  (0.619,0.937) | 0.853  (0.689,0.950) | 0.836  (0.736,0.918) | 0.948  (0.859,0.988) | 1.001 |
| FA-AD-RD | 0.885  (0.778,0.953) | 0.852  (0.663,0.958) | 0.912  (0.763,0.981) | 0.885  (0.800,0.951) | 0.965  (0.883,0.995) | 0.000 |
| FA-MD-RD | 0.770  (0.645,0.868) | 1.000  (0.872,1.000) | 0.588  (0.407,0.754) | 0.764  (0.648,0.867) | 0.964  (0.882,0.995) | 0.085 |
| FA-MD-AD-RD | 0.885  (0.778,0.953) | 0.852  (0.663,0.958) | 0.912  (0.763,0.981) | 0.885  (0.801,0.951) | 0.944  (0.854,0.987) | 1.062 |

Data in parentheses are 95% CIs.

**Table S3.** Sensitivity analysis of the proportion of target-domain data used for UDA training on model AUC

| **Model** | **Data proportion of UDA fitting sample** | | | |
| --- | --- | --- | --- | --- |
|  | **40%** | **60%** | **80%** | **100%** |
| FA-MD | 0.927 (0.855,0.970) | 0.936 (0.866,0.976) | 0.913 (0.838,0.961) | 0.942 (0.875,0.980) |
| FA-MD-AD | 0.874 (0.790,0.933) | 0.900 (0.821,0.952) | 0.866 (0.780,0.927) | 0.882(0.800,0.939) |
| FA-MD-RD | 0.954 (0.891,0.986) | 0.939 (0.870,0.978) | 0.956 (0.892,0.987) | 0.929 (0.858,0.972) |
| FA-MD-AD-RD | 0.836 (0.746,0.904) | 0.860 (0.773,0.922) | 0.890 (0.809,0.945) | 0.864 (0.778,0.926) |

The data used for the sensitivity analysis did not overlap with the target-domain test set.

**Table S4.** Performance of DenseNet model on target-domain test set

| **Target-domain test set – model without unsupervised domain adaptation** | | | | | | |
| --- | --- | --- | --- | --- | --- | --- |
| Diffusion scalar  image combinations | Accuracy | Recall | Specificity | F1 | AUC | z value of  DeLong test |
| FA-MD | 0.884  (0.802,0.941) | 0.889  (0.759,0.963) | 0.880  (0.757,0.955) | 0.884  (0.820,0.947) | 0.929  (0.857, 0.972) | 0.000 |
| FA-MD-AD | 0.789  (0.694,0.866) | 0.622  (0.465,0.762) | 0.940  (0.835,0.987) | 0.783  (0.696,0.862) | 0.856  (0.770,0.920) | 1.585 |
| FA-MD-RD | 0.853  (0.765,0.917) | 0.844  (0.705,0.935) | 0.860  (0.733,0.942) | 0.853  (0.779,0.926) | 0.937  (0.868,0.977) | 0.275 |
| FA-MD-AD-RD | 0.726  (0.625,0.813) | 0.489  (0.3370.642) | 0.940  (0.835,0.987) | 0.710  (0.606,0.806) | 0.756  (0.657,0.838) ⃰ | 2.944 |

Data in parentheses are 95% CIs. *: *p* value is significant (<0.05) when comparing AUC values with the best AUC in the same dataset using the DeLong test.

**Table S5.** Performance comparison of deep learning models

| **Internal test set** | | | | | | | |
| --- | --- | --- | --- | --- | --- | --- | --- |
| data combination | Model | Accuracy | Recall | Specificity | F1 | AUC | z value of DeLong test |
| FA-MD | DenseNet | **0.902**  (0.798,0.963) | **0.889**  **(0.708,0.976)** | 0.912  (0.763,0.981) | 0.902  (0.820,0.967) | 0.951  (0.863,0.990) | 0.765 |
| FA-MD | VGG11 | 0.787  (0.663,0.881) | 0.593  (0.388,0.776) | 0.941  (0.803,0.993) | 0.778  (0.658,0.882) | 0.953  (0.866,0.991) | 0.751 |
| FA-MD | ResNet18 | 0.836  (0.719,0.918) | 0.778  (0.577,0.914) | 0.882  (0.725,0.967) | 0.835  (0.739,0.918) | 0.915  (0.815,0.971) | 1.436 |
| FA-MD-RD | DenseNet | **0.902**  **(0.798,0.963)** | **0.889**  **(0.708,0.976)** | 0.912  (0.763,0.981) | **0.902**  (0.820,0.967) | **0.962**  (0.879,0.994) | 0.000 |
| FA-MD-RD | VGG11 | 0.754  (0.627,0.855) | 0.444  (0.255,0.647) | **1.000**  **(0.897,1.000)** | 0.729  (0.594,0.856) | 0.937  (0.844,0.983) | 1.270 |
| FA-MD-RD | ResNet18 | 0.754  (0.627,0.855) | 0.778  (0.577,0.914) | 0.735  (0.556,0.871) | 0.755  (0.643,0.867) | 0.861  (0.748,0.936)⃰ ⃰ | 2.609 |

Data in parentheses are 95% CIs. ⃰ ⃰ *p* value is significant (<0.01) when comparing AUC values with the best AUC using the DeLong test.

**Table S6.** Comparison of VCI imaging AI studies

| **Study** | **[38]** | **[39]** | **[40]** | **Ours** |
| --- | --- | --- | --- | --- |
| Model | VGG | ResNet | Vison transformer and  XGBoost | DenseNet |
| Modality | T2-FLAIR | T2-FLAIR | T1, T2-FLAIR,  clinical non-imaging data | DTI |
| Sample size  (Train / internal test / external test) | 197 / 66 / - | 194 / 48 / - | 246 / 61 / 157 | 244 / 61 / 72 |
| Multi-Center dataset | No | No | Yes | Yes |
| Domain generalization strategy | No | No | No | UDA |
| Analyses related to  cognitive domains or prognosis | No | No | No | Neuropsychological relevance analysis  of DenseNet and SSIM-based cognitive profiling |
| Model performance |  |  |  |  |
| Accuracy | 0.969 | 0.938 | - | 0.902 |
| F1 score | 0.930 | 0.942 | - | 0.902 |
| Internal test AUC | - | - | 0.972 (0.934,0.997) | 0.951 (0.863,0.990) |
| External test AUC | - | - | 0.902 (0.859,0.941) | 0.942 (0.875,0.980) |

UDA, unsupervised domain adaptation.

**Table S7.** SVCI identification model parameter summary

| Model | Parameter | Memory usage (MB) | epoch |
| --- | --- | --- | --- |
| Our DenseNet | 523,034 | 560.57 | 50 |
| Our DenseNet-UDA | 574,364 | 560.77 | 200 |
| VGG11 | 8,605,602 | 2715.33 | 50 |
| ResNet18 | 33,660,930 | 3093.32 | 70 |

DenseNet-UDA, DenseNet with unsupervised domain adaptation.

**Table S8**. Sensitivity analysis of SSIM across different MI maps construction

| **MMSE** |  |  |
| --- | --- | --- |
| **Cluster** | **SSIM (Full sample)** | **SSIM (80% subsample)** |
| Low risk | 0.344±0.011 | 0.476±0.010 |
| Moderate risk | 0.376±0.013 | 0.504±0.007 |
| High risk | 0.438±0.017 | 0.546±0.008 |
| Cluster change rate: 0.091 |  |  |
| Spearman correlation between full sample and subsample SSIM scores: 0.964 | | |
| **MoCA** |  |  |
| **Cluster** | **SSIM (Full sample)** | **SSIM (80% subsample)** |
| Low risk | 0.314±0.016 | 0.259±0.016 |
| Moderate risk | 0.361±0.017 | 0.300±0.014 |
| High risk | 0.431±0.019 | 0.389±0.031 |
| Cluster change rate: 0.318 |  |  |
| Spearman correlation between full sample and subsample SSIM scores: 0.899 | | |
| **Immediate recall** |  |  |
| **Cluster** | **SSIM (Full sample)** | **SSIM (80% subsample)** |
| Low risk | 0.443±0.006 | 0.412±0.006 |
| Moderate risk | 0.466±0.007 | 0.438±0.009 |
| High risk | 0.495±0.011 | 0.478±0.014 |
| Cluster change rate: 0.091 |  |  |
| Spearman correlation between full sample and subsample SSIM scores: 0.880 | | |
| **Delayed recall** |  |  |
| **Cluster** | **SSIM (Full sample)** | **SSIM (80% subsample)** |
| Low risk | 0.431±0.008 | 0.438±0.003 |
| Moderate risk | 0.453±0.005 | 0.459±0.007 |
| High risk | 0.484±0.009 | 0.495±0.007 |
| Cluster change rate: 0.250 |  |  |
| Spearman correlation between full sample and subsample SSIM scores: 0.971 | | |
| **TMT-A** |  |  |
| **Cluster** | **SSIM (Full sample)** | **SSIM (80% subsample)** |
| Low risk | 0.381±0.011 | 0.408±0.010 |
| Moderate risk | 0.416±0.010 | 0.439±0.009 |
| High risk | 0.476±0.017 | 0.493±0.012 |
| Cluster change rate: 0.000 |  |  |
| Spearman correlation between full sample and subsample SSIM scores: 0.996 | | |
| **TMT-B** |  |  |
| **Cluster** | **SSIM (Full sample)** | **SSIM (80% subsample)** |
| Low risk | 0.348±0.013 | 0.382±0.012 |
| Moderate risk | 0.385±0.014 | 0.416±0.010 |
| High risk | 0.451±0.011 | 0.476±0.018 |
| Cluster change rate: 0.114 |  |  |
| Spearman correlation between full sample and subsample SSIM scores: 0.994 | | |

Cluster change rate: the proportion of subjects whose cluster assignments changed when SSIM was recalculated using MI maps derived from the 80% subsample compared with the full sample MI maps.

REFERENCES

[1] Roh JH, Lee JH. Recent updates on subcortical ischemic vascular dementia. Journal of stroke, 2014, 16: 18-26

[2] Cannistraro RJ, Badi M, Eidelman BH*, et al.* Cns small vessel disease: A clinical review. Neurology, 2019, 92: 1146-1156

[3] Grau-Olivares M, Arboix A, Bartrés-Faz D*, et al.* Neuropsychological abnormalities associated with lacunar infarction. J Neurol Sci, 2007, 257: 160-165

[4] Jacova C, Pearce LA, Costello R*, et al.* Cognitive impairment in lacunar strokes: The sps3 trial. Annals of neurology, 2012, 72: 351-362

[5] Seo SW, Ahn J, Yoon U*, et al.* Cortical thinning in vascular mild cognitive impairment and vascular dementia of subcortical type. Journal of neuroimaging : official journal of the American Society of Neuroimaging, 2010, 20: 37-45

[6] Lin QQ, Chen HS, Yang Y*, et al.* Small vessel disease burden and prognosis of recent subcortical ischaemic stroke differ by parent artery atherosclerosis. European journal of neurology, 2024, 31: e16422

[7] Hussenoeder FS, Conrad I, Roehr S*, et al.* Mild cognitive impairment and quality of life in the oldest old: A closer look. Quality of life research : an international journal of quality of life aspects of treatment, care and rehabilitation, 2020, 29: 1675-1683

[8] Hamilton OKL, Backhouse EV, Janssen E*, et al.* Cognitive impairment in sporadic cerebral small vessel disease: A systematic review and meta-analysis. Alzheimer's & Dementia, 2021, 17: 665-685

[9] Sudo FK, Amado P, Alves GS*, et al.* A continuum of executive function deficits in early subcortical vascular cognitive impairment: A systematic review and meta-analysis. Dementia & neuropsychologia, 2017, 11: 371-380

[10] Sachdev PS, Bentvelzen AC, Kochan NA*, et al.* Revised diagnostic criteria for vascular cognitive impairment and dementia-the vascog-2-wso criteria. JAMA neurology, 2025, 82: 1103-1112

[11] Skrobot OA, Black SE, Chen C*, et al.* Progress toward standardized diagnosis of vascular cognitive impairment: Guidelines from the vascular impairment of cognition classification consensus study. Alzheimer's & dementia : the journal of the Alzheimer's Association, 2018, 14: 280-292

[12] Jia X, Wang Z, Huang F*, et al.* A comparison of the mini-mental state examination (mmse) with the montreal cognitive assessment (moca) for mild cognitive impairment screening in chinese middle-aged and older population: A cross-sectional study. BMC Psychiatry, 2021, 21: 485

[13] Roebuck-Spencer TM, Glen T, Puente AE*, et al.* Cognitive screening tests versus comprehensive neuropsychological test batteries: A national academy of neuropsychology education paper†. Archives of clinical neuropsychology : the official journal of the National Academy of Neuropsychologists, 2017, 32: 491-498

[14] Meier IB, Buegler M, Harms R*, et al.* Using a digital neuro signature to measure longitudinal individual-level change in alzheimer’s disease: The altoida large cohort study. npj Digital Medicine, 2021, 4: 101

[15] Ye Q, Bai F. Contribution of diffusion, perfusion and functional mri to the disconnection hypothesis in subcortical vascular cognitive impairment. Stroke and Vascular Neurology, 2018, 3: 131-139

[16] van den Brink H, Doubal FN, Duering M. Advanced mri in cerebral small vessel disease. International Journal of Stroke, 2022, 18: 28-35

[17] Loftus JR, Puri S, Meyers SP. Multimodality imaging of neurodegenerative disorders with a focus on multiparametric magnetic resonance and molecular imaging. Insights into Imaging, 2023, 14: 8

[18] Inoue Y, Shue F, Bu G*, et al.* Pathophysiology and probable etiology of cerebral small vessel disease in vascular dementia and alzheimer's disease. Molecular neurodegeneration, 2023, 18: 46

[19] Prins ND, Scheltens P. White matter hyperintensities, cognitive impairment and dementia: An update. Nature reviews Neurology, 2015, 11: 157-165

[20] Huang J, Cheng R, Liu X*, et al.* Unraveling the link: White matter damage, gray matter atrophy and memory impairment in patients with subcortical ischemic vascular disease. Front Neurosci, 2024, 18: 1355207

[21] Alber J, Alladi S, Bae H-J*, et al.* White matter hyperintensities in vascular contributions to cognitive impairment and dementia (vcid): Knowledge gaps and opportunities. 2019, 5: 107-117

[22] Wen W, Sachdev PS, Li JJ*, et al.* White matter hyperintensities in the forties: Their prevalence and topography in an epidemiological sample aged 44-48. Human brain mapping, 2009, 30: 1155-1167

[23] Fazekas F, Chawluk JB, Alavi A*, et al.* Mr signal abnormalities at 1.5 t in alzheimer's dementia and normal aging. AJR American journal of roentgenology, 1987, 149: 351-356

[24] de Vocht F. [health complaints and cognitive effects caused by exposure to mri scanner magnetic fields]. Tijdschrift voor diergeneeskunde, 2007, 132: 46-47

[25] Debette S, Bombois S, Bruandet A*, et al.* Subcortical hyperintensities are associated with cognitive decline in patients with mild cognitive impairment. Stroke, 2007, 38: 2924-2930

[26] Xu Q, Zhou Y, Li Y-S*, et al.* Diffusion tensor imaging changes correlate with cognition better than conventional mri findings in patients with subcortical ischemic vascular disease. Dementia and Geriatric Cognitive Disorders, 2010, 30: 317-326

[27] Jung N-Y, Han CE, Kim HJ*, et al.* Tract-specific correlates of neuropsychological deficits in patients with subcortical vascular cognitive impairment. Journal of Alzheimers Disease, 2016, 50: 1125-1135

[28] Ferris J, Greeley B, Yeganeh NM*, et al.* Exploring biomarkers of processing speed and executive function: The role of the anterior thalamic radiations. NeuroImage: Clinical, 2022, 36: 103174

[29] Nucifora PG, Verma R, Lee SK*, et al.* Diffusion-tensor mr imaging and tractography: Exploring brain microstructure and connectivity. Radiology, 2007, 245: 367-384

[30] Tae WS, Ham BJ, Pyun SB*, et al.* Current clinical applications of diffusion-tensor imaging in neurological disorders. Journal of clinical neurology (Seoul, Korea), 2018, 14: 129-140

[31] Patel B, Markus HS. Magnetic resonance imaging in cerebral small vessel disease and its use as a surrogate disease marker. International Journal of Stroke, 2011, 6: 47-59

[32] Qin Q, Qu J, Yin Y*, et al.* Unsupervised machine learning model to predict cognitive impairment in subcortical ischemic vascular disease. Alzheimers & Dementia, 2023, 19: 3327-3338

[33] Liu B, Meng S, Cheng J*, et al.* Diagnosis of subcortical ischemic vascular cognitive impairment with no dementia using radiomics of cerebral cortex and subcortical nuclei in high-resolution t1-weighted mr imaging. Frontiers in Oncology, 2022, 12:

[34] Li Z, Wu M, Yin C*, et al.* Machine learning based on the eeg and structural mri can predict different stages of vascular cognitive impairment. Frontiers in Aging Neuroscience, 2024, 16:

[35] Lin G, Chen W, Geng Y*, et al.* A multimodal mri-based machine learning framework for classifying cognitive impairment in cerebral small vessel disease. Scientific Reports, 2025, 15: 13112

[36] Ciulli S, Citi L, Salvadori E*, et al.* Prediction of impaired performance in trail making test in mci patients with small vessel disease using dti data. IEEE Journal of Biomedical and Health Informatics, 2016, 20: 1026-1033

[37] Diciotti S, Ciulli S, Ginestroni A*, et al.* Multimodal mri classification in vascular mild cognitive impairment. City: Year. 4278-4281

[38] Wang Y, Tu D, Du J*, et al.* Classification of subcortical vascular cognitive impairment using single mri sequence and deep learning convolutional neural networks. Frontiers in Neuroscience, 2019, 13:

[39] Chen Q, Wang Y, Qiu Y*, et al.* A deep learning-based model for classification of different subtypes of subcortical vascular cognitive impairment with flair. Frontiers in Neuroscience, 2020, 14:

[40] Fan F, Song H, Jiang J*, et al.* Development and validation of a multimodal deep learning framework for vascular cognitive impairment diagnosis. iScience, 2024, 27:

[41] Ganin Y, Lempitsky V. Unsupervised domain adaptation by backpropagation. City: PMLR, Year. 1180-1189

[42] Ahn E, Kumar A, Fulham M*, et al.* Unsupervised domain adaptation to classify medical images using zero-bias convolutional auto-encoders and context-based feature augmentation. IEEE Transactions on Medical Imaging, 2020, 39: 2385-2394

[43] Garcia-Dias R, Scarpazza C, Baecker L*, et al.* Neuroharmony: A new tool for harmonizing volumetric mri data from unseen scanners. NeuroImage, 2020, 220: 117127

[44] Huang G, Liu Z, Maaten LVD*, et al.* Densely connected convolutional networks. City: Year. 2261-2269

[45] Springenberg JT, Dosovitskiy A, Brox T*, et al.* Striving for simplicity: The all convolutional net. 2014,

[46] Zhang Q, Li J, Bian M*, et al.* Retinal imaging techniques based on machine learning models in recognition and prediction of mild cognitive impairment. Neuropsychiatric Disease and Treatment, 2021, 17: 3267-3281

[47] Wang Y, Lu P, Zhan Y*, et al.* The contribution of white matter diffusion and cortical perfusion pathology to vascular cognitive impairment: A multimode imaging-based machine learning study. Frontiers in Aging Neuroscience, 2021, 13:

[48] Pantoni L, Marzi C, Poggesi A*, et al.* Fractal dimension of cerebral white matter: A consistent feature for prediction of the cognitive performance in patients with small vessel disease and mild cognitive impairment. NeuroImage: Clinical, 2019, 24: 101990

[49] Chen H-J, Gao Y-Q, Che C-H*, et al.* Diffusion tensor imaging with tract-based spatial statistics reveals white matter abnormalities in patients with vascular cognitive impairment. Frontiers in Neuroanatomy, 2018, 12:

[50] Qiu Y, Yu L, Ge X*, et al.* Loss of integrity of corpus callosum white matter hyperintensity penumbra predicts cognitive decline in patients with subcortical vascular mild cognitive impairment. Frontiers in Aging Neuroscience, 2021, 13:

[51] Qin Q, Tang Y, Dou X*, et al.* Default mode network integrity changes contribute to cognitive deficits in subcortical vascular cognitive impairment, no dementia. Brain Imaging and Behavior, 2021, 15: 255-265

[52] Friston KJ. The disconnection hypothesis. Schizophrenia Research, 1998, 30: 115-125

[53] Qiao Y, He X, Zhang J*, et al.* The associations between white matter disruptions and cognitive decline at the early stage of subcortical vascular cognitive impairment: A case-control study. Front Aging Neurosci, 2021, 13: 681208

[54] Wardlaw JM, Smith EE, Biessels GJ*, et al.* Neuroimaging standards for research into small vessel disease and its contribution to ageing and neurodegeneration. The Lancet Neurology, 2013, 12: 822-838

[55] Chua TC, Wen W, Slavin MJ*, et al.* Diffusion tensor imaging in mild cognitive impairment and alzheimer's disease: A review. Curr Opin Neurol, 2008, 21: 83-92

[56] Lin L, Xue Y, Duan Q*, et al.* Microstructural white matter abnormalities and cognitive dysfunction in subcortical ischemic vascular disease: An atlas-based diffusion tensor analysis study. Journal of Molecular Neuroscience, 2015, 56: 363-370

[57] Ran W, Yu Q. Data-driven clustering approach to identify novel clusters of high cognitive impairment risk among chinese community-dwelling elderly people with normal cognition: A national cohort study. Journal of global health, 2024, 14: 04088
